# The Microbial Metabolite IDA Sensitizes Tumor Cells to ARF1 Inhibition by Inducing SVIL-dependent Senescence

**DOI:** 10.7150/ijbs.133716

**Published:** 2026-08-24

**Authors:** Menglin Huang, Xuan Wang, Yanfeng Jiang, Jincheng Li, Binliu Hu, Yuetong Wang, Steven X. Hou

**Affiliations:** Department of Cell and Developmental Biology at School of Life Sciences, State Key Laboratory of Genetics and Development of Complex Phenotypes, Institute of Metabolism and Integrative Biology, Zhongshan Hospital, Fudan University, Shanghai 200438, China.

**Keywords:** microbial metabolite, cellular senescence, trans-3-indoleacrylic acid, ARF1 inhibitor, M1-like macrophage

## Abstract

While pharmacological inhibition of ARF1 exhibits robust antitumor activity, its therapeutic efficacy is constrained by tissue-specific limitations. To augment the clinical potential of our developed ARF1 inhibitor, DU102, we pursued a strategy of senescence induction. A screen for endogenous senescence-inducing metabolites identified trans-3-indoleacrylic acid (IDA), a microbiota-derived metabolite, as a potent senescence inducer. We demonstrate that IDA acts through targeting supervillin (SVIL), leading to subsequent enhancement of the expression of the senescence-associated enzyme galactosidase beta 1 (GLB1). *In vivo*, IDA enhanced the antitumor efficacy of DU102 through senescence-dependent sensitization, overcoming tissue-restricted resistance and achieving potent tumor suppression. Further analysis revealed that this combination remodels the tumor microenvironment by coordinately regulating tumor-associated macrophages through different predominant mechanisms: IDA predominantly promotes M1-like polarization via TNFα, whereas ARF1 inhibition primarily drives macrophage expansion through CSF2 upregulation. Our work unveils a novel metabolite-regulated senescence pathway and establishes a rational combination therapy that effectively co-opts tumor-associated macrophages (TAMs) for cancer treatment.

## Introduction

ADP-ribosylation factor 1 (ARF1), a small GTPase traditionally known for its roles in vesicle trafficking and lipid homeostasis^1^, has recently been identified as a significant driver of tumorigenesis^2^, ^3^. Its overexpression is correlated with tumor progression in multiple cancer types, including ovarian cancer^4^, prostate cancer^5^, and head and neck squamous cell carcinoma (HNSCC)^6^, highlighting its potential as a therapeutic target. Despite inhibition of ARF1 demonstrates broad anti-tumor effects^6^-^8^, the clinical translation of existing inhibitors has been hampered by insufficient potency and specificity. In previous work, we identified the compound designated as 'DU102' as a potent ARF1 inhibitor that enhances immune-mediated tumor clearance across multiple models^9^.

Despite its potent antitumor activity, the therapeutic application of the ARF1 inhibitor DU102 is constrained by tissue-specific limitations, prompting the exploration of rational combination strategies. Induction of cellular senescence, a stable cell cycle arrest with context-dependent tumor-suppressive or tumor promotional effects, represents a promising therapeutic avenue^10^, ^11^. However, the efficacy of pro-senescence therapy is often counterbalanced by the persistence of senescent cells and their senescence-associated secretory phenotype (SASP)^12^, ^13^. Notably, senescent cells exhibit heightened susceptibility to proteostatic stress^14^, particularly upon disruption of coatomer protein complex I (COP I)-dependent trafficking, a process governed by ARF1^15^, ^16^. While COP I inhibition is known to exacerbate proteostatic imbalance and amplify SASP, it remains unclear whether this vulnerability can be therapeutically co-opted. Thus, a critical question persists: Can a pro-senescence therapy be effectively combined with ARF1 inhibition to trigger robust and sustained antitumor immunity?

To address this, we conducted a functional screen for endogenous metabolites that could induce senescence and sensitize tumor cells to ARF1 inhibition. Intriguingly, our screen identified a well-known gut microbiota-derived tryptophan metabolite, trans-3-indoleacrylic acid (IDA), as a candidate. IDA is known to mediate ferroptosis resistance through the canonical aryl hydrocarbon receptor (AHR) signaling pathway^17^-^19^. Despite its established metabolic identity, its potential to act as a direct inducer of cellular senescence and to enhance the sensitivity of tumor cells to targeted molecular therapies like Arf1 inhibition, has remained entirely unexplored.

Here, we report that IDA can induce cellular senescence and enhance the antitumor efficacy of DU102 to achieve robust tumor suppression. We further elucidate that this effect is mechanistically grounded in a concerted reshaping of the tumor-associated macrophages landscape. IDA functions by targeting the actin-binding protein supervillin (SVIL) to establish a senescent state that instructs M1-like macrophage polarization through TNFα, while DU102 acts in a complementary but partially distinct manner by upregulating colony stimulating factor 2 (CSF2) to expand the macrophage pool.

## Results

### IDA induces senescence and sensitizes tumor cells to DU102 for enhanced antitumor efficacy

To evaluate the efficacy of DU102 in eliminating senescent cells, we treated Pan02 cells with etoposide to induce senescence^20^ (**Fig. S1A-D**) and then administered DU102. The combination of etoposide and DU102 showed a detectable antitumor effect *in vitro* (**Fig. S1E-F**), confirming that senescent cells become more responsive to DU102. Furthermore, DU102 treatment significantly alleviated bleomycin-induced pulmonary fibrosis, a senescence-associated model^21^ (**Fig. S1G-H**), suggesting that DU102 may eliminate the senescent cells *in vivo*.

However, the modest efficacy of etoposide in our combination regimen and its inherent toxicity presented a significant limitation^22^, while the endogenous metabolites could serve as safer senescence inducers. Thus, we performed an endogenous metabolite library screening using the p21-RFP reporter system in Panc02 cells (murine pancreatic ductal adenocarcinoma) to identify compounds capable of triggering senescence (**Fig. 1A-B**). Among the identified candidates, IDA robustly induced cellular senescence and showed the greatest antitumor efficacy when combined with DU102 (**Fig. 1C-D and Fig. S2A-C**). Moreover, IDA effectively induced senescence and showed enhanced antitumor effect when combined with DU102 across multiple cancer cell lines, including LLC, Hepa1-6 and Panc02, as demonstrated by senescence-associated beta-galactosidase (SA-β-gal) staining and crystal violet staining (**Fig. 1E-F**). In the presence of IDA, the half maximal inhibitory concentration (IC_50_) of DU102 was significantly reduced in these cells (**Fig. 1G**). To further evaluate the translational relevance, we extended our analysis to human cancer cell lines A549 and Panc1, where the combination of IDA and DU102 also showed enhanced antitumor activity in crystal violet assays (**Fig. S3A-B**).

To determine whether this enhanced activity reflects classical synergy, we performed Chou-Talalay analysis. It did not show strong synergy at the low concentrations used for senescence induction in our regimen (e.g., IDA 50-100 μM, corresponding to Fa ≈ 0.05-0.15), indicating that the enhanced efficacy at these concentrations is not attributable to classical synergy (**Fig. S3C-E**). We therefore hypothesized that the enhanced efficacy at senescence-inducing concentrations reflects senescence-dependent sensitization rather than classical synergy. To test this, we pretreated cells with IDA to induce senescence, removed IDA, and then measured the IC_50_ of DU102. IDA-pretreated Panc02 and A549 cells showed a significantly reduced the IC_50_ of DU102 compared to untreated controls (**Fig. S3F-G**), indicating that the senescent state itself, rather than the concurrent presence of IDA, is sufficient to sensitize tumor cells to DU102. 293T cells, which are SV40 large T antigen-immortalized human embryonic kidney epithelial cells with a functionally inactive p53 axis^23^, ^24^, are refractory to IDA-induced senescence (**Fig. S3I**). Accordingly, IDA pretreatment did not reduce the IC50 of DU102 in this cell line (**Fig. S3H**), further supporting a senescence-dependent mechanism. Additionally, co-treatment with rapamycin, an mTOR inhibitor known to suppress senescence phenotypes^25^, ^26^, abrogated IDA-induced senescence markers (e.g., p-p53, p21) and prevented the reduction in DU102 IC_50_ (**Fig. S3J-M**), confirming that the sensitizing effect is senescence-dependent. Collectively, these data demonstrate that IDA sensitizes tumor cells to DU102 through a senescence-dependent mechanism, rather than through simple additivity or classical synergy.

Moreover, the combination of IDA and DU102 significantly enhanced tumor suppression in C57BL/6 mouse subcutaneous xenograft models, including Panc02 (**Fig. 2A-C**) and KPT, a cell line derived from KP mouse (LSL-*Kras*^G12D/+^; LSL-*Trp53*^R172H/+^; LSL-*Cas9*-EGFP) lung adenocarcinoma (**Fig. S4B-D**)*.* And SA-β-gal staining further confirmed that IDA treatment could induce senescence *in vivo*, while the combination with DU102 markedly reduced SA-β-gal positivity, consistent with senescent cell elimination by DU102 (**Fig. S4A**). Importantly, no significant body weight loss or major organ damage was observed in KPT tumor-bearing mice treated with the combination, as assessed by H&E staining of heart, liver, spleen, lung, and kidney (**Fig. S4E-F**). These results indicate that IDA acts as a senescence inducer and enhances the antitumor efficacy of DU102.

### IDA enhances DU102 antitumor efficacy in the lung through modulation of local microbiota

Although DU102 can reshape the tumor microenvironment and shows promise as an anticancer agent^9^, its effect exhibits context-dependent efficacy across different tissues. DU102 treatment significantly inhibited the orthotopic liver tumor progression (**Fig. 2D-E**), while it had no significant antitumor effect in the orthotopic lung cancer model under the same dosing regimen (**Fig. 2F-G**). As expected, combined treatment with IDA and DU102 significantly suppressed tumor growth in the orthotopic lung cancer model (**Fig. 2F-G**), indicating that IDA can enhance DU102 efficacy in a tissue-restricted context and expand the clinical application scope of DU102.

Given that IDA is a tryptophan-derived indole metabolite produced by gut microbiota^17^, we investigated whether local microbial composition contributes to the tissue-specific response to DU102. 16S rRNA profiling of microbial communities from the colon, lung, and liver of C57BL/6 mice revealed that *Peptostreptococcales*, an order that includes species (e.g., *Peptostreptococcus anaerobius*) known to produce IDA, was enriched in the colon^19^. In contrast, the *Burkholderiales*, a Gram-negative bacteria order known to efficiently degrade indole compounds through specific enzymatic pathways^27^-^30^, was dominated specifically in the lung (**Fig. 2H**). Given this metabolic capability, we suspected that local *Burkholderiales* might also process and inactivate IDA, thereby limiting DU102's efficacy in the lung. To assess this, we depleted these bacteria using a combination of metronidazole (Met) and neomycin (Neo) (**Fig. 2I**)^31^. This antibiotic combination is known to primarily target Gram-negative bacteria, which include the order *Burkholderiales*^31^. Consistent with our hypothesis, this treatment enhanced the ability of DU102 to suppress tumor growth in the orthotopic lung cancer model (**Fig. 2J-K**). Furthermore, untargeted metabolomic analysis revealed that IDA was undetectable in lung tissues under steady-state conditions but became readily detectable after antibiotic treatment (**Fig. 2L**), supporting the notion that local Gram-negative bacteria, potentially including members of the order *Burkholderiales*, contribute to IDA metabolism. Clinically, the appearance of *Burkholderiales* in the gut microbiota is also correlated with higher cancer incidence in the TaiZhou cohort (**Fig. 2M**). Together, these observations suggest that local Gram-negative bacteria may modulate DU102 efficacy in the lung by affecting IDA availability.

### Tumor-associated macrophages are required for the anti-tumor efficacy of IDA

To identify the immune cell population responsible for the anti-tumor effects of IDA, we first treated tumor-bearing NCG (NOD/ShiLtJGpt-Prkd^cem26Cd52^Il2rg^em26Cd22^ Hr^em1Cin8936^/Gpt) mice, which lack functional T, B, and NK cells. Consistent with our observations in immunocompetent wild-type (WT) mice, IDA significantly suppressed tumor growth (**Fig. 3A-C**), indicating that its efficacy is independent of adaptive immunity.

Senescence-associated secretory phenotype (SASP) is the major feature of senescent cells^32^-^34^. Thus, we hypothesized that the IDA treatment might activate innate immune cells via altering the secretion of immunomodulatory factors of tumor cells. By SASP expression analysis, we found that IDA treatment modulated the expression of several immunomodulatory factors in Panc02 cells, including *Ccl2*, *Ccl7*, *Il10*, and *Isg15* (**Fig. S5A**), several of which have been implicated in macrophage biology. To determine whether macrophages are functionally involved in IDA's antitumor activity, we utilized *Lyz2*-DTR mice, in which monocyte/macrophage lineage can be conditionally depleted upon diphtheria toxin (DT) treatment (**Fig. S5B**)^35^, ^36^. In mice without DT treatment, IDA treatment effectively inhibited tumor growth (**Fig. 3D-F**), while this function was largely diminished in DT-treated mice (**Fig. 3G-I**). Flow cytometric analysis confirmed that DT treatment efficiently depleted the total macrophage pool following DT administration (**Fig. 3J-K**). Notably, in mice from the control group, IDA treatment significantly reduced total macrophage numbers while increasing the proportion of activated M1-like macrophages, marked by CD86⁺ positive (**Fig. 3J-M**). These results demonstrate that macrophages are essential for the anti-tumor activity of IDA.

We further analyzed the role of macrophages in the combined treatment of IDA and DU102. In the LLC subcutaneous model, flow cytometry analysis revealed that DU102 treatment increased the infiltration of total macrophages and, more notably, the increased frequency of CD80^+^CD86^+^ macrophages within tumors was further enhanced in the combination treatment group (**Fig. 3N-Q**), suggesting that the combined treatment robustly promoted the activation of M1 macrophages while it decreased M2 macrophages. Furthermore, we performed IHC staining on lung sections from the antibiotic-treated orthotopic lung cancer model. Tumor-infiltrated M1-like macrophages were significantly increased in the mice of either antibiotic or DU102 treatment group, while in the combined group the tumor infiltrated M1-like macrophages could hardly be quantified due to barely any tumors (**Fig. S5C-D**). Together, these data suggested that macrophages are vital for the function of IDA treatment and are closely associated with the enhanced efficacy of the combination treatment.

### IDA and DU102 modulate tumor-associated macrophages through partially distinct mechanisms

Given the established critical role of macrophages in mediating the antitumor effects of IDA and its combination with DU102 *in vivo*, we sought to delineate the specific contributions of each drug to macrophage activation. We first examined the polarization state of RAW264.7 macrophages exposed to conditioned medium (CM) from Panc02 cells pretreated with each drug. Quantitative PCR analysis revealed that CM from Panc02 cells treated with IDA broadly upregulated expression of classic M1-like macrophage markers (*Nos2, Cxcl10, Tnfa, Il1b*) and downregulated M2 markers (*Arg1, Tgfb, Il4*), whereas DU102 CM showed a more modest effect, primarily upregulating *Cxcl10* and downregulating *Il4* and *Il13* (**Fig. 4A**). Consistent with these findings, Western blot analysis confirmed that IDA CM increased IL-1β protein levels while reducing ARG1 expression (**Fig. 4B**). Together, these results indicate that IDA primarily drives M1-like polarization, whereas DU102 exerts a more limited effect on polarization markers.

We next assessed functional responses using a transwell migration assay. Macrophages co-cultured with IDA-pretreated Panc02 cells exhibited a reduced migratory capacity and displayed an activated morphology characterized by enlarged cell size and extended protrusions (**Fig. 4C**). Conversely, co-culture with DU102-pretreated cells promoted a robust migratory and proliferative morphology, with macrophages appearing more numerous and rounded. The combination of both drugs resulted in a more pronounced activated macrophage phenotype (**Fig. 4C**). These observations suggest that IDA primarily induces M1 polarization, which may be associated with reduced proliferation/migration, while DU102 predominantly enhances macrophage recruitment and expansion.

To quantitatively validate these findings, we performed flow cytometric analysis of macrophages cultured with the different CM. Consistent with the morphological data, CM from IDA-pretreated Panc02 cells led to an overall reduction in the total number of macrophages (**Fig. 4D**). However, the proportion of CD80⁺CD86⁺ cells within the macrophage gate was increased (**Fig. 4E, S6A**). Notably, IDA CM also reduced the proportion of CD163⁺CD206⁺ M2-like macrophages **(Fig. 4G, S6B)**, resulting in a net increase in the M1/M2 ratio **(Fig. 4H)**. This pattern indicates that IDA enriches for M1-like macrophages and reduces the M2 population, thereby shifting the overall balance toward a pro-inflammatory phenotype, even as total macrophage numbers decline. In contrast, CM from DU102-pretreated Panc02 cells markedly increased the numbers of total macrophages (**Fig. 4D**), suggesting that DU102 primarily expands the macrophage pool. Critically, the combination treatment integrated both effects, resulting in a significant increase in both the absolute number and the proportion of CD80⁺CD86⁺ M1 macrophages, thereby further elevating the M1/M2 ratio (**Fig. 4D-H**). To assess the functional consequence of this polarization shift, we performed a phagocytosis assay. Macrophages pretreated with the indicated CM showed significantly enhanced engulfment of mCherry-expressing CT26 tumor cells upon IDA CM treatment, whereas DU102 CM had a more modest effect (**Fig. 4I-J**). These data indicate that IDA not only shifts the M1/M2 balance but also enhances the functional phagocytic activity of macrophages.

To identify the key soluble factors responsible for the observed macrophage remodeling, we profiled the secretome of drug-treated Panc02 cells and focused on cytokines associated with macrophage polarization and proliferation. IDA treatment alone caused a modest elevation in the secretion of polarization-related factors including *Tgfb* and *Il10*, but strikingly upregulated *Tnfa* and *Cxcl10* while downregulating *Il4* (**Fig. 5A**). In contrast, DU102 treatment significantly upregulated the expression of colony stimulating factor 2 (*Csf2/GM-Csf*). The combination of IDA and DU102 led to a broad and significant upregulation of both polarization (e.g., *Tnfa*) and proliferation/recruitment-associated factors (e.g., *Csf1*, *Csf2*, *Ccl2*) (**Fig. 5A**). These results were consistent with our initial hypothesis that IDA primarily provides a polarizing signal, while DU102 predominantly induces a proliferative/recruiting signal in tumor cells. Thus, we generated *Csf2*-knockdown Panc02 cells (**Fig. S7A**). Indeed, *Csf2* knockdown significantly reduced the production of key polarization cytokines (**Fig. 5B**). Functional transwell assays demonstrated that, unlike CM from DU102-treated control cells, CM from DU102-treated *Csf2* knockdown cells failed to enhance macrophage migration and proliferation (**Fig. 5C**). Furthermore, *Csf2* knockdown substantially attenuated the activated macrophage morphology (e.g., enlarged size, pale staining) and diminished the increase in M1 macrophage numbers induced by the combination treatment (**Fig. 5C**). Notably, the knockdown of *Csf2* also impaired the response to IDA treatment. In *Csf2* knockdown cells, IDA treatment no longer significantly reduced the total number of macrophages nor increased the proportion of CD80⁺CD86⁺ macrophages, indicating a blunted polarizing capacity (**Fig. S7B-E**). Consistently, *Csf2* knockdown also markedly reduced the phagocytic activity of macrophages in response to IDA-treated tumor cell-CM, as assessed by a phagocytosis assay (**Fig. S7F-G**). Additionally, *Csf2* knockdown in Panc02 subcutaneous xenografts significantly compromised the antitumor efficacy of the combination treatment of IDA and DU102 compared to wild-type tumors, as demonstrated by increased tumor size, volume, and weight (**Fig. 5D-F**). Flow cytometric analysis further revealed that neither DU102 monotherapy nor the combination treatment significantly increased the proportion of F4/80⁺CD11b⁺ macrophages or the frequency of CD80⁺CD86⁺ M1 macrophages within the tumor microenvironment in *Csf2*-knockdown tumors (**Fig. 5G-J**). Collectively, these findings demonstrate CSF2 as a key mediator of DU102-induced macrophage expansion and an essential factor for the enhanced anti-tumor activity of DU102 and IDA.

In a parallel investigation, we assessed the role of TNFα, a key polarization cytokine induced by IDA. Knockdown of *Tnfa* in tumor cells impaired the ability of IDA-conditioned medium to induce M1 polarization in macrophages, as evidenced by blunted upregulation of M1 markers (*Il1b, Ccl5, Il6*) and a concomitant lack of suppression (or even an increase) in M2-associated markers (*Il10*, *Tgfb*) (**Fig. S8A-C**). This suggests that TNFα contributes to the polarizing function of IDA. Collectively, these data demonstrate that IDA-driven M1 macrophage differentiation and DU102-mediated expansion of the macrophage pool cooperatively increase the tumor-suppressive M1 population within the tumor microenvironment.

### IDA induces senescence by targeting SVIL

To comprehensively understand the molecular mechanisms by which IDA induces tumor cell senescence, we performed immunoprecipitation-mass spectrometry (IP-MS) through labeling IDA to the magnetic beads to identify potential protein targets. Among the top candidates, PRRC2C, SVIL, and FN1 were identified (**Fig. 6A**). We therefore examined the functional relevance of these candidates by assessing senescence marker expression upon IDA treatment in cells with individual knockdown of each candidate. Notably, knockdown of *Svil* most profoundly attenuated IDA-induced senescence, as indicated by the failure of IDA treatment to further increase senescence marker expression beyond the basal level observed in *Svil*-knockdown cells (**Fig. 6B-C, S9A-C**). To further validate the interaction between IDA and SVIL, we performed Drug Affinity Responsive Target Stability (DARTS) assays using SVIL-FLAG protein obtained by anti-FLAG pull-down. Consistently, IDA treatment protected SVIL-FLAG from thermolysin-induced degradation compared to the vehicle control, suggesting that IDA binding confers stability to SVIL (**Fig. 6D**).

Given the evidence that SVIL can translocate to the nucleus and function as a transcriptional coactivator^37^-^39^, we hypothesized that IDA interacts with SVIL and recruits it to the promoter of the senescence-associated gene *Glb1*. To test this, we performed chromatin immunoprecipitation (ChIP) assays using an anti-SVIL antibody in Panc02 cells, followed by qPCR analysis of the *Glb1* promoter region. IDA treatment markedly enhanced the enrichment of SVIL at the *Glb1* promoter compared to untreated cells (**Fig. 6E**). Notably, the region between -1476 and -1381 bp relative to the transcription start site (TSS) exhibited the highest fold enrichment upon IDA treatment (**Fig. 6F**). We further validated this enrichment in Hepa1-6 cells, focusing on the most responsive regions (**Fig. S9D**). In contrast, in 293T cells, SVIL protein levels were substantially lower and IDA treatment failed to enhance SVIL enrichment at the *Glb1* promoter (**Fig. S9E-F**), consistent with the lack of senescence induction in this cell line. These results suggest that IDA promotes SVIL association with *Glb1* promoter, thereby activating its transcription and contributing to the senescence phenotype.

Moreover, in subcutaneous xenografts derived from control cells, IDA treatment significantly reduced tumor burden, as evidenced by decreased tumor size, volume, and weight (**Fig. 6G-I**). However, this antitumor effect was completely abolished in xenografts derived from *Svil***-**knockdown cells (**Fig. 6L-N**). Consistent with this, SA-β-gal staining of tumor sections revealed that IDA failed to enhance senescence in *Svil***-**knockdown xenografts compared to controls (**Fig. 6Q**). Furthermore, flow cytometric analysis of tumor-infiltrating immune cells showed that IDA treatment in shluc tumors decreased the proportion of F4/80⁺CD11b⁺ macrophages among live cells, while increasing the CD80⁺CD86⁺ M1-like subset within the macrophage population, consistent with our previous findings (**Fig. 6J-K, S9G**). In contrast, these immunomodulatory effects were largely absent in sh*Svil* tumors (**Fig. 6O-P, S9H**), indicating that SVIL is also required for IDA-mediated remodeling of the tumor immune microenvironment. Collectively, these findings identify SVIL as a key mediator of IDA-induced senescence and macrophage modulation.

To determine whether the modulation of macrophage polarization by IDA treatment depends on targeting SVIL-mediated senescence, we examined the effects of conditioned medium (CM) from control and *Svil*-knockdown cells on macrophage function. We compared the effects of CM from control and *Svil*-knockdown cells. CM from *Svil*-knockdown cells induced a cytokine secretion profile similar to that of IDA-treated cells, including altered levels of *Tnfa*, *Cxcl10*, and *Il13* (**Fig. 7A**), suggesting that *Svil* depletion itself can mimic IDA in promoting macrophage differentiation. Flow cytometry analysis further demonstrated that CM from IDA-treated control cells robustly induced a shift toward M1-like macrophages, as previously reported (**Fig. 7C-F**). However, CM from IDA-treated *Svil*-knockdown cells failed to significantly reduce macrophage numbers or consistently enhance the M1 subset (**Fig. 7C-F**), demonstrating a markedly attenuated effect and indicating that the immunomodulatory function of IDA treatment is dependent on SVIL. Transwell assays confirmed that *Svil*-knockdown also reduced macrophage migration, similar to the effect of IDA treatment (**Fig. 7B**). Additionally, a phagocytosis assay revealed that CM from IDA-treated control cells enhanced macrophage engulfment of mCherry-expressing tumor cells, whereas CM from IDA-treated *Svil*-knockdown cells showed markedly reduced phagocytic activity (**Fig. 7G-H**). Collectively, these *in vitro* data demonstrate that SVIL is essential for IDA-induced senescence and its downstream immunomodulatory effects on macrophages, as *Svil* knockdown alone establishes a basal senescent state that cannot be further enhanced by IDA.

To further assess whether *Svil* knockdown phenocopies the immunomodulatory effect of IDA treatment, we examined macrophage polarization markers following treatment with CM from DU102-exposed cells. CM from *Svil*-knockdown cells treated with DU102 induced a macrophage phenotype resembling that caused by the IDA and DU102 combination, characterized by elevated expression of M1 markers (*Nos2*,* Cxcl10*, *Tnfa*) together with unchanged or reduced levels of M2-associated genes (*Arg1*, *Tgfb*) (**Fig. 7I**). These results indicate that SVIL protein is the key target mediating IDA immunomodulatory effects, and enables DU102 to promote an M1-like macrophage polarization state.

To explore the clinical relevance of SVIL, we analyzed its expression in TCGA datasets. In bladder urothelial carcinoma (BLCA), mesothelioma (MESO), and ovarian cancer (OV), high SVIL expression was significantly associated with poor overall survival (**Fig. S9I**). This is consistent with published reports showing that elevated SVIL expression correlates with unfavorable prognosis in endometrial cancer^40^, ovarian cancer^41^. These observations suggest that SVIL may serve as a prognostic biomarker in these cancer types. Whether SVIL expression levels could predict response to IDA-based therapy warrants further investigation.

## Discussion

Senolytic represents a promising anticancer strategy; however, its translation is often impeded by challenges such as poor bioavailability, low target specificity, and a lack of validated biomarkers^13^, ^42^-^45^. Targeting ARF1-mediated vesicle trafficking has been reported as a potential approach for senescence-based therapy.

However, a critical challenge to realizing this therapeutic synergy is the identification of robust senescence inducers that are both effective and exhibit favorable combinatory potential with Arf1-targeted therapy. While exogenous compounds like CDK4/6 and mTOR inhibitors can induce senescence^46^-^48^, their broad mechanisms often lead to pleiotropic effects and poor specificity^49^-^51^. Furthermore, the landscape of endogenous molecules capable of intrinsically triggering senescence remains largely unexplored^34^. We have previously reported an independently developed ARF1 inhibitor, DU102. In this study, to expand the clinical application and optimize the therapeutic effect, we performed endogenous metabolite screening and identified the microbial metabolite IDA as a novel senescence inducer. Further functional study demonstrates that its combination with the ARF1 inhibitor DU102 not only enhances antitumor activity but effectively overcomes the limited efficacy of DU102 in the lung.

IDA was previously characterized as a tumor-promoting agent that confers resistance to ferroptosis in colon cancer models^17^-^19^. In stark contrast, our data demonstrate a potent tumor-suppressive effect of IDA in our lung cancer and pancreatic cancer models, mediated through binding and enhancing SVIL-regulated GLB1 transcription. This apparent paradox underscores the context-dependent nature of metabolic signaling in cancer and suggests that the ultimate impact of IDA on tumor fate may be determined by the specific cellular state or microenvironment. Our previous work has shown that ARF1 modulates the gut microbiota^52^, providing a biological basis for the tissue-dependent efficacy of DU102. Considering that IDA is a microbiota-catabolized tryptophan metabolite and that ARF1 modulates the gut microbiota, the tissue-specific presence of *Peptostreptococcales* and* Burkholderiales*, may contribute to the complex function of IDA and the differential efficacy of DU102 across tissues. While our antibiotic approach offers a valuable initial clue, future studies employing germ-free mice colonized with defined bacterial strains are needed to establish causality. It should be noted that the antibiotics used in our study primarily target Gram-negative bacteria; therefore, the restored DU102 efficacy upon antibiotic treatment cannot be attributed solely to the depletion of *Burkholderiales*. Additionally, we cannot formally exclude the possibility that DU102 itself, which also contains an indole moiety, might be metabolized by gut or local bacteria. However, DU102 is a synthetic compound with a rigid polycyclic scaffold distinct from simple indole derivatives, and our preliminary characterization indicates that it is highly stable *in vivo*, making such metabolism unlikely.

SVIL has long been recognized as an ACTIN-binding protein with diverse functions in cytoskeletal organization, membrane dynamics, and transcriptional regulation. Previous studies have implicated SVIL in modulating androgen receptor activity and suppressing p53-mediated cell death, suggesting its potential role in promoting cell survival and tumor progression^39^. However, our work uncovers a novel function of SVIL as a key mediator of cellular senescence, wherein IDA binding facilitates SVIL recruitment to the senescence-associated gene *Glb1*. Through chromatin immunoprecipitation assays, we confirmed that IDA specifically enhances SVIL enrichment at the *Glb1* promoter, particularly within the -1476 to -1381 bp region relative to the transcription start site, correlating with the activation of *Glb1* expression and the initiation of the senescence program. This mechanism is further supported by functional studies showing that SVIL knockdown not only attenuates IDA-induced senescence but also diminishes IDA's antitumor efficacy *in vivo*, highlighting the non-redundant role of SVIL in mediating IDA's biological effects. Notably, *Svil* knockdown alone elicited a basal level of senescence, suggesting that SVIL may function as a homeostatic regulator; however, the mechanism underlying this basal senescence requires further investigation. Several questions remain to be addressed in future studies. First, the structural basis of the IDA-SVIL interaction warrants investigation to determine whether IDA binds directly to SVIL's actin-binding domain, transcriptional activation domain, or another regulatory region, and how this binding modulates SVIL's subcellular localization and cofactor interactions. In addition, formal validation of the functional relevance of SVIL occupancy at the *Glb1* promoter (e.g., via promoter mutagenesis) awaits future investigation. Second, the upstream signaling pathways that regulate SVIL expression and activity in cancer cells need to be elucidated, as targeting these pathways could potentiate the efficacy of IDA or related compounds. Additionally, the clinical relevance of the IDA-SVIL-*Glb1* axis merits exploration, particularly whether SVIL expression levels or genetic variations in the *Glb1* promoter region correlate with cancer patient prognosis or response to the combined therapies.

Cellular senescence is a double-edged sword. While acute senescence induced by oncogenic stress or certain therapeutic agents can suppress tumor growth in the short term, persistent senescent cells may create a pro-inflammatory microenvironment that paradoxically promotes tumor relapse, metastasis, or immune evasion through the senescence-associated secretory phenotype (SASP). Our study focused on relatively short-term endpoints (tumor growth and survival up to 35 days post-implantation). Therefore, the long-term consequences of IDA-induced senescence remain to be determined.

Our secretome analysis further revealed that *Csf2* knockdown reduces the expression of multiple polarization-related cytokines (*Tnfa*, *Il4*, *Il10*) in Panc02 cells, indicating that CSF2 is required for maintaining the basal expression of these factors. This suggests an autocrine-supportive role of CSF2, rather than a specific effect on IDA-induced senescence, consistent with reports that GM-CSF can act as an autocrine factor in tumor cells^53^ and that senescent cells secrete GM-CSF to influence tumor cell behavior^54^. Although our study did not directly test the molecular pathway, these precedents support the idea that CSF2 may act back on pancreatic cancer cells to sustain the expression of a broad cytokine program. Whether this involves CSF2 receptor signaling, transcriptional cooperation with IDA-induced pathways, or post-transcriptional regulation remains to be determined.

Moreover, our mechanistic study revealed that IDA and DU102 coordinately modulate the tumor immune microenvironment. IDA acts as a “polarizing instructor,” targeting the SVIL to trigger a senescent state and an associated secretome that promotes M1-like macrophage polarization. Whereas, DU102 serves as a “myeloid expander,” upregulating CSF2 to amplify the total macrophage pool. This cooperative action significantly increases tumor-suppressive M1 macrophages population within the tumor microenvironment. It is important to note that although our study focuses on macrophages, the full antitumor efficacy of DU102, as well as the combined treatment, likely involves broader immune engagement, including activating anti-tumor T-cell immunity^9^.

In summary, our work establishes a new therapeutic paradigm that combines senescence induction with ARF1 inhibition, achieving potent antitumor efficacy and paving the way for senolytic-based combination strategies.

## Material and Methods

### Experimental model and subject details

#### Mice

KP mice, conditionally expressing mutant Kras^G12D^ and Trp53^fl/fl^ along with a LSL-Cas9-EGFP reporter, were generated through sequential crossing of B6.129S4-*Kras^tm4Tyj^*/J, B6.129P2-*Trp53^tm1Brn^*/J, and B6;129-*Gt* (*ROSA*) *26Sor^tm1^*^(CAG-cas9*,-EGFP)*Fezh*^/J. *Lyz2*-DTR mice were generated by crossing C57BL/6-*Gt(ROSA)26Sor^tm1(HBEGF)Awai^*/J with B6.129P2-*Lyz2^tm1(cre)Ifo^*/J. All mice used in this study were 6-8 weeks of age at the start of experiments. For subcutaneous tumor models, C57BL/6J (WT) mice were inoculated with 0.8 × 10⁶ Panc02 cells, 0.8 × 10⁶ Panc02-shCSF2 cells, 0.5 × 10⁶ KPT cells, or 0.5 × 10⁶ LLC cells, while NCG mice were engrafted solely with 0.8 × 10⁶ Panc02 cells. Drug treatment was initiated when tumors reached a palpable size of approximately 3×4 mm. Drugs were prepared by dissolution in DMSO followed by dilution in corn oil (final DMSO concentration 5% v/v) and administered via daily intraperitoneal injection (DU102 at 5 mg/kg, IDA at 50 mg/kg, or DMSO control). All animal experiments were performed in accordance with the guidelines approved by the Institutional Animal Care and Use Committee of the Institute of Developmental Biology and Molecular Medicine (IDMIACUC), Fudan University, under protocol number IDM20240706.

#### Lung cancer model

In the spontaneous lung tumor model, KP mice received intranasal administration of Cre-recombinase adenovirus to initiate tumorigenesis, followed by a two-week microbiota modulation regimen involving weekly intranasal antibiotics (1 mg/mL metronidazole and neomycin) and continuous antibiotics in drinking water until the endpoint, prior to intermittent drug treatment (See Fig. 2K).

#### Myeloid cell depletion model

For myeloid cell depletion studies, Lyz2-DTR mice underwent a defined diphtheria toxin (DT) regimen administered before and after Panc02 inoculation, with drug treatment coinciding with the third DT pulse (See Fig. S3C).

#### Liver cancer model

For the MYC-ON liver cancer model, mice fed a doxycycline-containing diet were switched to a normal diet at 6 weeks of age. After inducing liver tumor for 2 weeks, mice were treated with Arf1 inhibitors for 6 weeks and were euthanized with CO2 after 5 weeks.

#### Pulmonary fibrosis model

For the pulmonary fibrosis model, wild type C57BL6 mice were treated with 2.5mg/kg bleomycin via nasal inhalation and analyzed 21days after treatment.

#### Cells

##### Drug Treatment

Cells were seeded and allowed to adhere for 12-24 hours prior to drug treatment. To induce senescence, tumor cells were treated with IDA (50μM) for 5 days. Following this preconditioning, both IDA-induced senescent cells and control cells were plated separately and subjected to a subsequent 24-hour drug treatment. DU102 was used at 5 μM unless otherwise indicated.

##### Establishment of stable knockdown cell lines

Lentiviral particles were produced by co-transfecting HEK293T cells with the shRNA transfer plasmid, psPAX2, and pMD2.G using EZ Transfection Reagent. Medium was replaced 8-12 hours post-transfection. Viral supernatants collected at 48 and 72 hours were pooled, clarified by centrifugation at 4400 × g for 5 min, and used to transduce target tumor cells. After 24-48 hours, viral medium was replaced with fresh complete medium. Puromycin selection was initiated 48 hours post-transduction to establish stable knockdown pools.

### Method details

#### Hematoxylin and eosin (H&E) staining

Mouse lungs were perfused, and liver tissues were fixed with 4% paraformaldehyde (PFA). After washing with PBS, the tissues were dehydrated through a graded ethanol series, cleared in xylene, and embedded in paraffin. Sections were cut, baked dry, and then deparaffinized in xylene followed by rehydration through a graded ethanol series to water. The sections were stained with hematoxylin, rinsed under running water, counterstained with eosin, dehydrated through another graded ethanol series, and finally mounted with neutral resin.

#### Immunohistochemistry (IHC) staining

Paraffin-embedded sections were baked dry, deparaffinized in xylene, and rehydrated through a graded ethanol series to water. Endogenous peroxidase activity was quenched by incubation with 3% hydrogen peroxide for 15 minutes at room temperature. Antigen retrieval was performed by microwaving the sections in 1× citrate buffer (5 min on high, 2 min for thawing, and 20 min on medium-low power), followed by cooling naturally to room temperature. After washing, the sections were incubated with Reagent A from the SPlink Detection Kits for 15 minutes at room temperature. Subsequently, the sections were incubated with a primary antibody (anti-CD68, anti-iNOS) overnight. Following PBS washes, the sections were sequentially incubated with Reagent B and Reagent C from the SPlink Detection Kits (each for 15 minutes at room temperature, with PBS washes in between). Color development was achieved using a DAB kit. Finally, the sections were counterstained with hematoxylin, dehydrated, air-dried, and mounted with neutral resin.

#### Lung fibrosis assessment

For lung fibrosis assessment, mice were treated with bleomycin (2.5 mg/kg) via intratracheal instillation on day 0. On day 21, lungs were harvested, fixed in 4% paraformaldehyde, and embedded in paraffin. Sections (5 μm) were stained with Masson's trichrome according to the manufacturer's instructions (Sigma-Aldrich). Collagen deposition (blue area) was quantified using ImageJ software. For each mouse, five randomly selected fields per section were analyzed, and the percentage of blue area relative to the total lung parenchyma area was calculated. The averaged values were used for statistical comparison.

#### Senescence-associated β-galactosidase (SA-β-gal) staining

SA-β-gal staining was performed using the commercial kit listed in the Materials. For cell samples, staining was conducted following a 24-hour drug treatment. For tumor tissues, samples were fixed in 4% PFA, cryoprotected in 30% sucrose, embedded in OCT compound, and sectioned at 8μm thickness. All samples (both cells and tissue sections) were then processed for SA-β-gal activity according to the manufacturer's protocol. Following staining, the sections were mounted with AntiFade Mounting Medium for microscopic observation.

#### Cell Counting Kit-8 (CCK-8) assay

For IDA pretreatment experiments, cells were first treated with IDA (50 μM) for 5 days to induce senescence. The culture medium was then removed, and cells were washed three times with PBS to eliminate residual drug. Cells were then trypsinized, reseeded into 96-well plates, and allowed to adhere for 12 h. Subsequently, cells were treated with a gradient of concentrations of DU102 (0-50 μM) for 48-72 h. Cell viability was assessed by measuring the absorbance at 450 nm after the addition of CCK-8 reagent. Data were normalized to vehicle-treated controls and presented as dose-response curves. IC_50_ values were calculated using a four-parameter logistic (4PL) model in GraphPad Prism.

For rapamycin rescue experiments, cells were treated with IDA (50 μM) and/or rapamycin (10 μM) for 5 days. The culture medium was then removed, and cells were washed three times with PBS to eliminate residual drugs. Cells were then trypsinized, reseeded into 96-well plates, and allowed to adhere for 12 h, followed by treatment with a gradient of DU102 concentrations for 48-72 h. Cell viability was measured and analyzed as described above.

#### Chou Talalay analysis

To determine the combination index, the IC_50_ of each drug was first measured in each cell line. A fixed molar ratio of DU102 to IDA was then chosen based on their respective IC_50_ values, and serial dilutions were prepared accordingly. Cells were treated for 48-72 h, and cell viability was assessed by CCK-8 assay. CI values were calculated using CompuSyn software according to the Chou-Talalay method.

#### Conditioned medium (CM) preparation

Tumor cells were seeded and treated with IDA (50 μM), DU102 (5 μM), their combination, or vehicle (DMSO) for 24 h. The culture medium was then carefully removed, and cells were washed three times with PBS to eliminate any residual drug. Fresh drug-free medium was added, and cells were incubated for an additional 24 h. The supernatant was collected, centrifuged at 2,000 × g for 10 min to remove cell debris, and stored at 4°C for immediate use. For macrophage treatment, CM was diluted with fresh culture medium at a ratio of 500 μL CM to 700 μL fresh medium prior to use.

#### Macrophage treatment with CM

RAW264.7 macrophages were serum-starved with Opti-MEM for 8 h. The medium was then replaced with the diluted CM (prepared as described above) and incubated for 72 h, with CM replenished every 24 h.

#### Phagocytosis assay

RAW264.7 macrophages were seeded in 6-well plates and serum-starved with Opti-MEM for 8 h. The medium was then replaced with the indicated conditioned medium (CM), and macrophages were treated for 72 h, with CM replenished every 24 h. After CM removal, cells were gently washed three times with PBS without disturbing the cell monolayer. mCherry-expressing CT26 tumor cells were then added to the macrophages and co-incubated for 3 h at 37 °C in a 5% CO₂ incubator. Following co-incubation, non-ingested tumor cells were removed by gently washing three times with PBS. Macrophages were then harvested by gentle pipetting and subjected to flow cytometric analysis. mCherry-expressing CT26 cells alone (without macrophages) were used as a negative control for gating. The percentage of mCherry⁺ cells within the CD11b⁺F4/80⁺CD80⁺CD86⁺ macrophage population was quantified to assess phagocytic activity.

#### Transwell migration assay

Transwell migration assay was performed using 8.0μm pore membrane inserts. Tumor cells were seeded in the lower chambers, treated with drugs for 24 hours, and then washed with PBS. Fresh culture medium was added to the lower chambers, and RAW 264.7 macrophages were subsequently seeded into the upper chambers to initiate a 72-hour co-culture. When tumor cells in any one of the treatment groups reached ~95% confluence, all macrophage-containing inserts from every group were simultaneously transferred to new wells pre-seeded with fresh, sub-confluent tumor cells. This ensured a continuous and potent chemotactic gradient across all experimental conditions throughout the assay.

#### Crystal violet staining

Cells were washed with 1× PBS and fixed with 4% PFA for 20 minutes at room temperature. Following fixation, samples were washed with 1× PBS and stained with a 1:10 dilution of crystal violet saturated methanolic solution for 10-20 minutes. Unbound dye was removed by thorough washing with distilled water until the background was clear. For Transwell migration assays, non-migrated cells on the upper surface of the membrane were carefully removed with a cotton swab prior to the final washing step. Stained samples were examined microscopically, with Transwell membranes kept immersed in water for observation.

#### IP-MS

Cell lysis buffer contained 25 mmol/L Tris-HCl (pH 7.4), 150 mmol/L NaCl, 1% NP-40, 1 mmol/L EDTA, 5% glycerol, and protease inhibitor. Cells were washed with PBS and lysed with the above buffer on ice. The cell lysates were transferred to a 1.5 mL centrifuge tube, and centrifuged at 13,000rpm for 10 min. The supernatant was transferred to a new centrifuge tube. Then the equal amount of protein was used in the IP experiment. Blank beads and IDA-labeled beads (Sangon, D601013) were added into the lysates and incubated overnight at 4 C. The beads were washed three times, resuspended in protein loading buffer, and finally examined by immunoblotting. Protein bands were visualized with Omni-ECL™Femto Light Chemiluminescence Kit (Epizyme, Cat# SQ201L) on BIO-RAD ChemiDoc Touch (America).

#### Chromatin immunoprecipitation followed by quantitative PCR (ChIP-qPCR)

The ChIP-qPCR assay was performed using the commercial kit listed in the Materials. Cells were seeded, allowed to adhere for 12-24 hours, and then treated for 24 hours with either DMSO or IDA. Subsequently, sample pretreatment was carried out following the kit manufacturer's protocol. Chromatin fragmentation was achieved using a Q800R focused ultrasonicator under the following conditions: 50% amplitude, 45 min total duration, with cycles of 20s on and 40s off. Immunoprecipitation with anti-SVIL/anti-IgG antibody, reverse cross-linking, and DNA purification were subsequently performing following the kit protocol. The enrichment of *Glb1* promoter region was subsequently quantified by qPCR.

#### Pull-down coupled with Drug Affinity Responsive Target Stability (DARTS) assay

To validate the direct interaction between IDA and SVIL, a pull-down coupled with DARTS assay was performed. 293T cells were transiently transfected with pcDNA-SVIL-FLAG plasmid to express FLAG-tagged SVIL protein. Cells were cultured in 10 cm dishes to 80-85% confluence, then washed three times with ice-cold PBS and lysed in NP-40 lysis buffer (containing 1× protease inhibitor cocktail and 1× phosphatase inhibitor cocktail) on ice for 10 min. Cell lysates were collected using a cell scraper and centrifuged at 18,000 × g for 10 min at 4°C. The supernatant was collected and incubated with anti-FLAG affinity beads overnight at 4°C with gentle rotation. After incubation, the beads were washed three times with lysis buffer and resuspended in a suitable volume of lysis buffer.

DARTS assay was performed following established protocols^55^. The purified SVIL-FLAG protein was divided into four aliquots and treated with either DMSO (vehicle) or IDA at the indicated concentration for 30 min at room temperature in the dark with gentle rotation. The samples were then diluted with 1× TNC buffer (10 mM Tris-HCl pH 8.0, 50 mM NaCl, 10 mM CaCl₂) to a final 1× concentration. Thermolysin (dissolved in 1× TNC buffer) was added to the designated samples at a predetermined optimal dilution (typically 1:400-1:2000), while control samples received an equal volume of 1× TNC buffer without protease. The mixtures were incubated at room temperature for 5-10 min with gentle rotation. Proteolysis was terminated by the addition of 2× volume of 50× protease inhibitor cocktail (final concentration ~1×). Samples were then mixed with 5× SDS-loading buffer, heated at 70°C for 10 min, and analyzed by Western blot using anti-FLAG antibody. Actin served as a loading control.

#### Flow cytometric analysis

For cultured cells, samples were washed with PBS, detached using 0.25% trypsin, and collected by centrifugation. For tumor tissues, single-cell suspensions were prepared by mechanical dissociation through a 70μm cell filter. All samples were subsequently washed with PBS and incubated with fluorochrome-conjugated antibodies diluted in Cell Staining Buffer (1:500 for other antibodies; 1:1000 for DAPI) for 45-60 minutes at room temperature in the dark. After staining, cells were washed with CSB, resuspended, and analyzed by flow cytometry.

#### Gene expression analysis

Total RNA was extracted from cells. Briefly, cells were washed with 1× PBS and homogenized in TRIzol reagent. RNA was precipitated with isopropanol, washed with 75% ethanol, and briefly air-dried before being dissolved in RNase-free water. cDNA was synthesized from 2 μg of DNase-treated total RNA using the NovoScript® Plus All-in-one 1st Strand cDNA Synthesis SuperMix (gDNA Purge). Quantitative PCR was performed with ChamQ SYBR qPCR Master Mix. Relative mRNA levels were normalized and presented as 2^-ΔΔCT^ value.

#### Survival analysis

Survival analysis was conducted via GEPIA (http://gepia.cancer-pku.cn), which integrates TCGA expression profiles and clinical follow-up data. Patients were dichotomized by the median expression of the target gene. Kaplan-Meier curves and log-rank tests were used to compare overall survival (OS) and progression-free interval (PFI). A two-sided p-value ≤ 0.05 was considered statistically significant.

### Quantification and statistical analysis

Please refer to the figure legends for experimental details, including descriptions of the samples (cells, mice) and statistical details. The number of mice used is shown in the figure legends. Data were plotted and analyzed using GraphPad Prism 9.0 software (GraphPad Software). Data are shown as the mean ± SEM (standard error of the mean). For comparisons between two groups, two-tailed unpaired Student's t-tests were used. For experiments involving more than two groups, one-way analysis of variance (ANOVA) with Tukey's post-hoc test was applied. When multiple groups were compared to a single control group, Dunnett's post-hoc test was used. For non-normally distributed data, the Kruskal-Wallis test with Dunn's multiple comparisons test was applied. A p-value ≤ 0.05 was considered statistically significant.

## Supplementary Material

Supplementary figures and table.

## Figures and Tables

**Figure 1:**
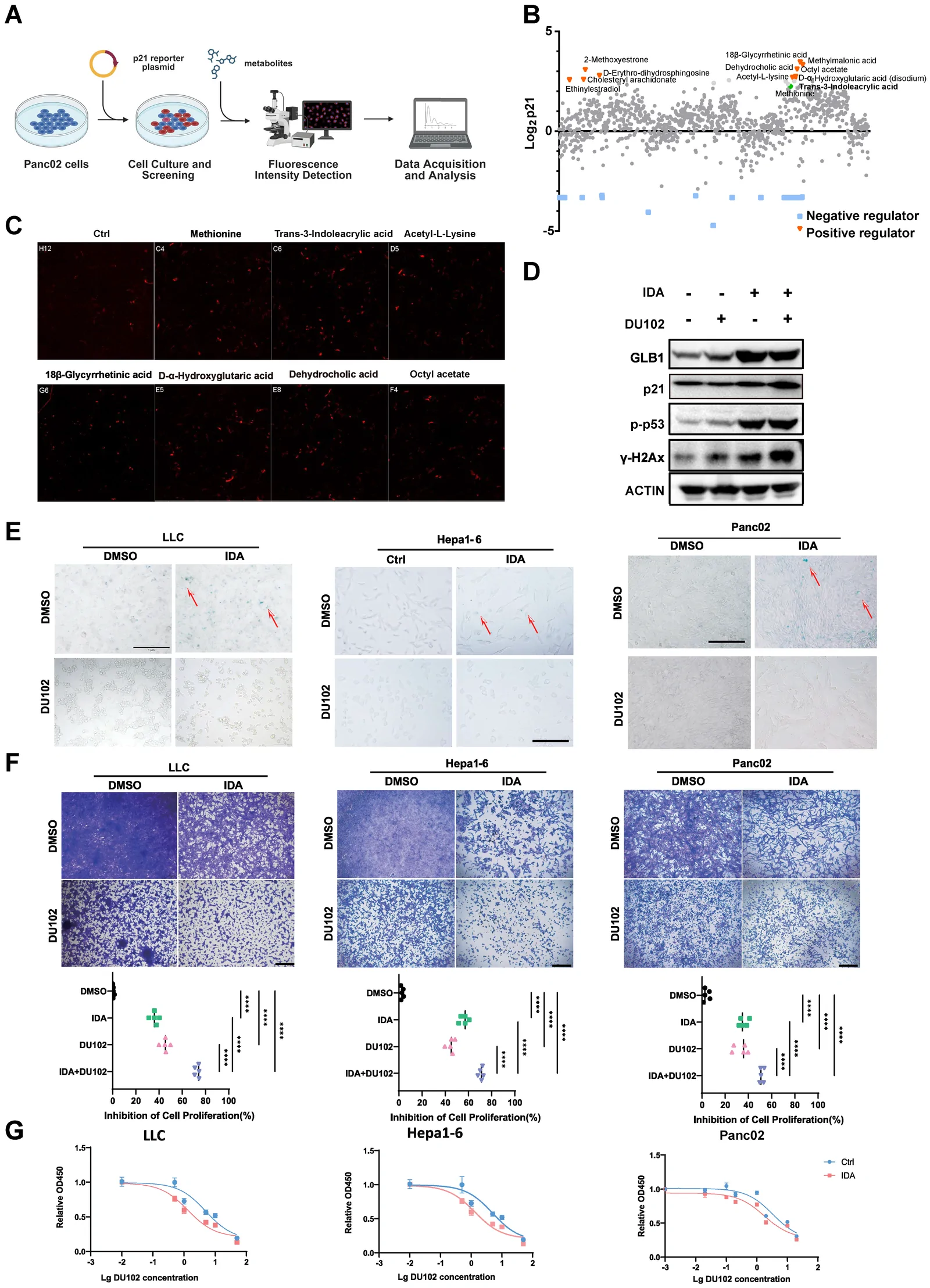
** Trans-3-Indoleacrylic acid induces senescence and sensitizes tumor cells to DU102 *in vitro*. (A)**Schematic diagram of the screening strategy for potential endogenous metabolites that induce senescence. **(B)** Scatter plot of log_2_-fold change in RFP fluorescence intensity (reporting p21 expression) in stable Panc02 p21-RFP cells treated with a library of endogenous metabolites, compared to DMSO control. Metabolites are categorized as positive or negative regulators of p21 based on log₂-transformed p21 expression.** (C)** Representative fluorescence microscopy images of Panc02 p21-RFP reporter cells treated with selected candidate metabolites from (B). Scale bar, 200µm. **(D)** Western blot analysis of senescence markers in Panc02 cells treated with DMSO, IDA (50 µM), DU102 (5µM), or their combination. ACTIN serves as a loading control.** (E)** SA-β-gal staining in Hepa1-6, LLC, and Panc02 cells after treatment as in (D). Scale bar, 1µm. **(F)** Crystal violet staining in Hepa1-6, LLC, and Panc02 cells after treatment as in (D) (n=5). Scale bar, 500µm. **(G)** Dose-response curves of cell viability (CCK-8 assay) after 24-hour treatment with increasing concentrations of DU102, alone or with fixed-dose IDA (50 µM) (n=3). Data are presented as mean ± SEM. Statistical significance was determined by one-way ANOVA with Tukey's multiple comparisons test (F). Only significant comparisons are indicated by asterisks (*p < 0.05; **p < 0.01; ***p < 0.001; ****p < 0.0001); non-significant comparisons are not indicated.

**Figure 2:**
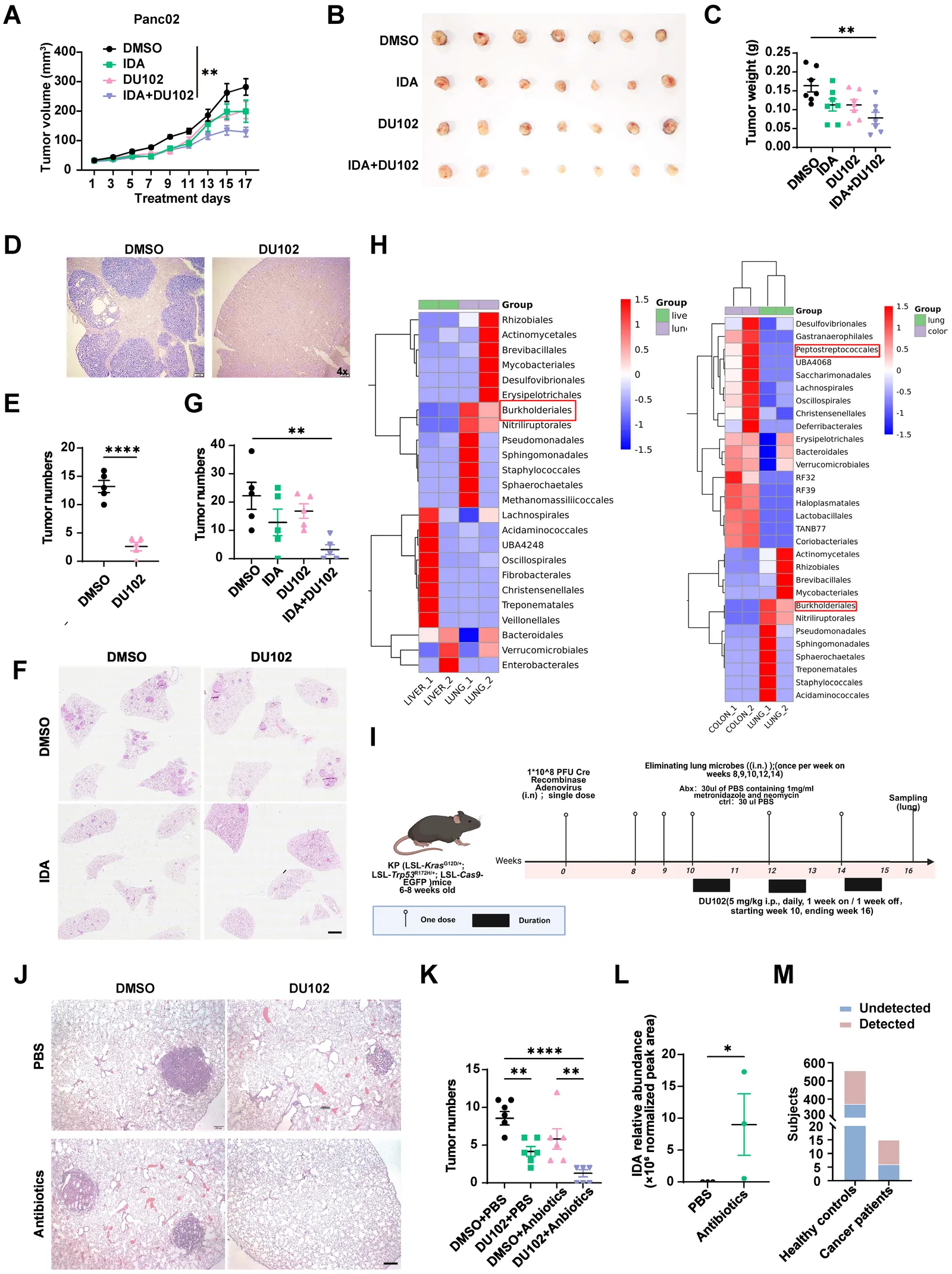
** IDA enhances DU102 efficacy in the lung in a microbiota-associated manner. (A-C)** C57BL/6 mice bearing subcutaneous Panc02 allografts were treated with DMSO, IDA (50 mg/kg), DU102 (5 mg/kg), or their combination (n=7). **(A)** Tumor growth curves, **(B)** final tumor volumes and** (C)** tumor weights at the study endpoint are shown. **(D-E)** Mice bearing orthotopic liver tumors were treated with DMSO or DU102 (5 mg/kg) (n=5). **(D)** Representative H&E-stained lung sections and **(E)** quantification of tumor foci per section are shown. Scale bar, 250μm.** (F-G)** Mice bearing orthotopic lung tumors were treated with DMSO, IDA (50mg/kg), DU102 (5 mg/kg), or their combination (n=5). **(F)** Representative H&E-stained lung sections and **(G)** quantification of tumor foci per section are shown. Scale bar, 1000μm.** (H)** Heatmaps of microbial community composition in lung, colon, and liver samples from C57BL/6 mice. **(I-L)** Microbiota depletion in KP mice bearing orthotopic lung tumors.** (I)** Schematic of the experimental design. Mice were pretreated with broad-spectrum antibiotics (Abx) or PBS (control), followed by DMSO or DU102 (5 mg/kg) treatment (n=6) **(J)** Representative H&E-stained lung sections and **(K)** quantification of tumor foci per section are shown. Scale bars, 250μm. **(L)** Relative abundance of IDA in lung tissues before and after antibiotics treatment, determined by untargeted LC-MS metabolomics (n=3). The detection limit was defined as a minimum peak height of 10,000; samples with signal intensity below this threshold were considered below the limit of detection (BDL) and are shown as open circles. **(M)**Mosaic plot showing the relative abundance of *Burkholderiales* in healthy controls versus tumor patients. Data are presented as mean ± SEM. Statistical significance was determined by two-way ANOVA with Tukey's multiple comparisons test (A), one-way ANOVA with Tukey's multiple comparisons test (C, G, K), or unpaired two-tailed t-test (E, G). Only significant comparisons are indicated by asterisks (*p < 0.05; **p < 0.01; ***p < 0.001; ****p < 0.0001); non-significant comparisons are not indicated.

**Figure 3:**
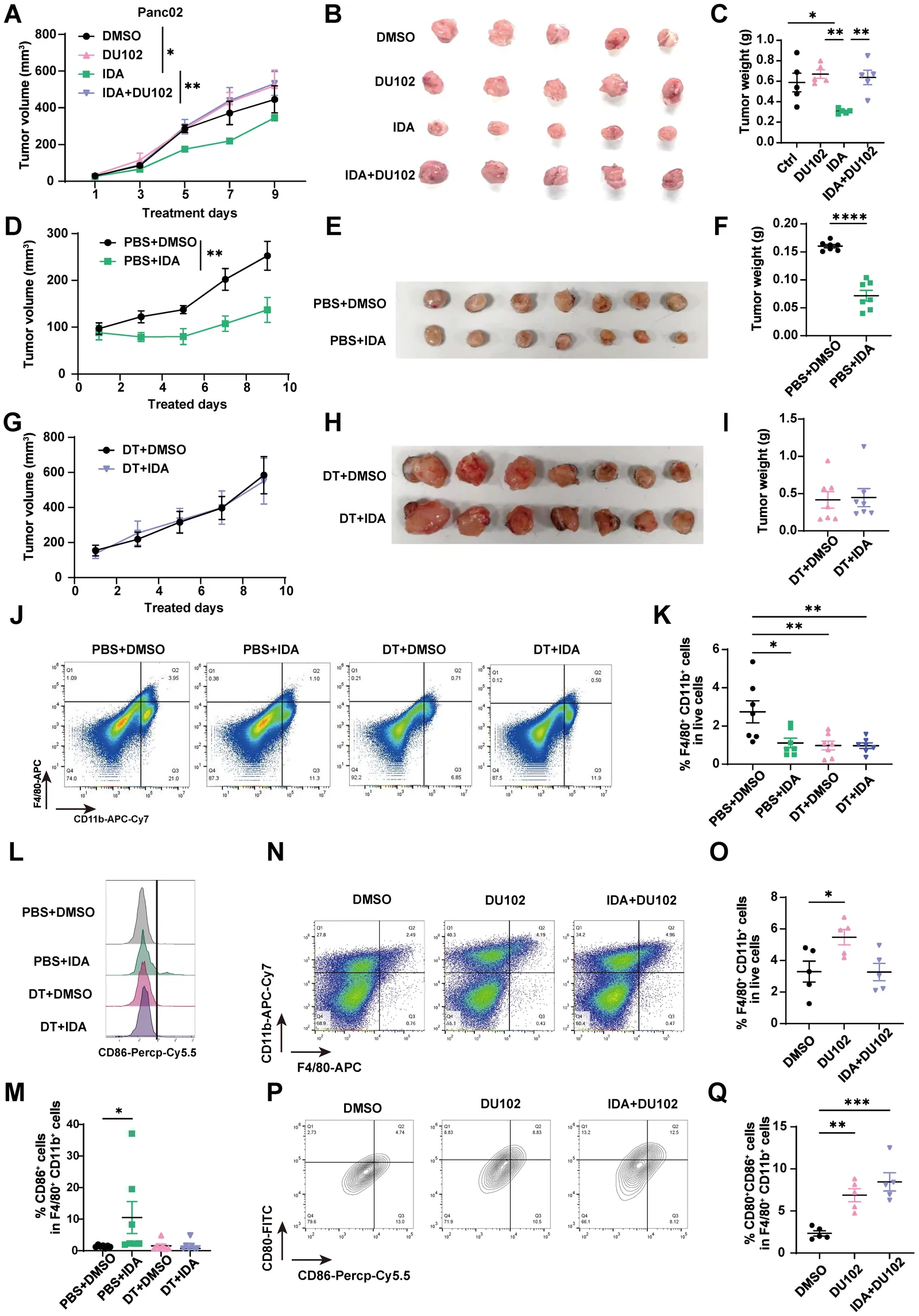
** Macrophages are required for the antitumor effect of IDA and DU102 combination therapy. (A-C)** NCG mice bearing subcutaneous Panc02 allografts were treated with DMSO, IDA (50 mg/kg), DU102 (5 mg/kg), or their combination (n=5). **(A)** Tumor growth curves, **(B)** final tumor volumes and** (C)** tumor weights at the study endpoint are shown.** (D-M)** *Lyz2*-DTR mice bearing subcutaneous Panc02 allografts were treated with PBS (control) or diphtheria toxin (DT) to deplete Lyz2^+^ cells, followed by drug administration (n=7). **(D)** tumor growth curves, **(E)** final tumor volumes, and **(F)** tumor weights at the study endpoint in PBS-treated control groups. **(G)** tumor growth curves,** (H)** final tumor volumes, and **(I)** tumor weights at the study endpoint in DT-treated groups. **(J)** Representative flow cytometry plots and **(K)** quantification of tumor-infiltrating F4/80⁺CD11b⁺ macrophages. **(L)** Representative flow cytometry histograms of CD86 expression and **(M)** quantification of CD86⁺ cells within the F4/80⁺CD11b⁺ macrophage population.** (N-Q)** Flow cytometric analysis of macrophage populations in LLC allografts from C57BL/6 mice treated with DMSO, DU102(5 mg/kg), or the combination of IDA (50 mg/kg) and DU102 (n=5).** (N)** Representative flow cytometry plots and **(O)** quantitation of F4/80⁺CD11b⁺ macrophages in live cells. **(P)** Representative flow cytometry plots and **(Q)** quantitation of CD80⁺CD86⁺ cells within the F4/80⁺CD11b⁺ macrophage population. Data in all panels are presented as mean ± SEM. Statistical significance was determined by Two-way ANOVA with Tukey's multiple comparisons test (A), one-way ANOVA with Tukey's multiple comparisons test (C, K, M, O, Q), or unpaired two-tailed t-test (D, F, G, I). Only significant comparisons are indicated by asterisks (*p < 0.05; **p < 0.01; ***p < 0.001; ****p < 0.0001); non-significant comparisons are not indicated.

**Figure 4:**
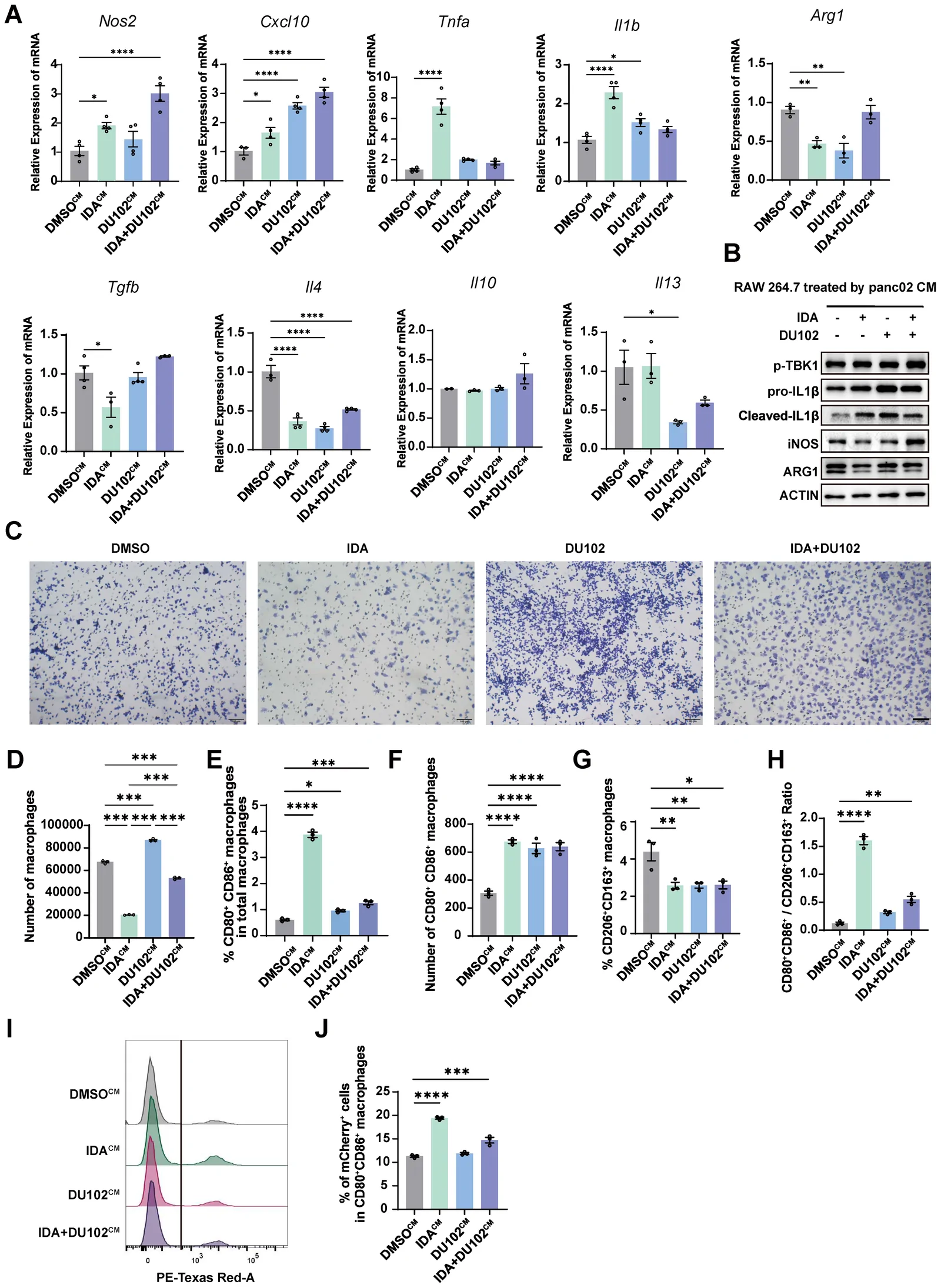
** IDA induces tumor cell senescence to modulate macrophage polarization. (A)** qPCR analysis of macrophage activation markers in RAW264.7 cells after 72-hour culture with conditioned medium (CM) from Panc02 cells pre-treated with DMSO, IDA (50μM), DU102 (5μM), or their combination. Data are normalized to *Gapdh* and presented as fold change relative to the DMSO-CM group (n=3). (**B**)Western blot analysis of macrophage activation markers in RAW264.7 cells after treatment with conditioned medium (CM) from Panc02 cells pre-treated as indicated. β-ACTIN served as a loading control.** (C)** Transwell migration assay of RAW264.7 cells towards drug pretreated Panc02 cells as in (A). Scale bar, 100 µm. **(D-H)** Flow cytometric analysis of RAW264.7 cells treated for 72 hours with CM as in (A). **(D)** Total cell counts of macrophages. **(E)** Proportion of CD80⁺CD86⁺ cells within the macrophage population. **(F)** Absolute number of CD80⁺CD86⁺ macrophages.** (G)** Proportion of CD163⁺CD206⁺ cells within the macrophage population.** (H)** Ratio of CD80⁺CD86⁺ M1-like to CD163⁺CD206⁺ M2-like macrophages.** (I-J)** Phagocytosis assay. RAW264.7 macrophages were pretreated with the indicated CM for 72 h, then co-cultured with mCherry-expressing CT26 tumor cells for 3 h. **(G)** Representative flow cytometry histogram of mCherry fluorescence within the CD11b⁺F4/80⁺CD80⁺CD86⁺ macrophage gate. **(H)** Quantification of the percentage of mCherry⁺ cells within the CD11b⁺F4/80⁺CD80⁺CD86⁺ macrophage population (n=3). Data in all panels are presented as mean ± SEM. Statistical significance was determined by one-way ANOVA with Tukey's multiple comparisons test (A, D-H, J), or unpaired two-tailed t-test (B). Only significant comparisons are indicated by asterisks (*p < 0.05; **p < 0.01; ***p < 0.001; ****p < 0.0001); non-significant comparisons are not indicated.

**Figure 5:**
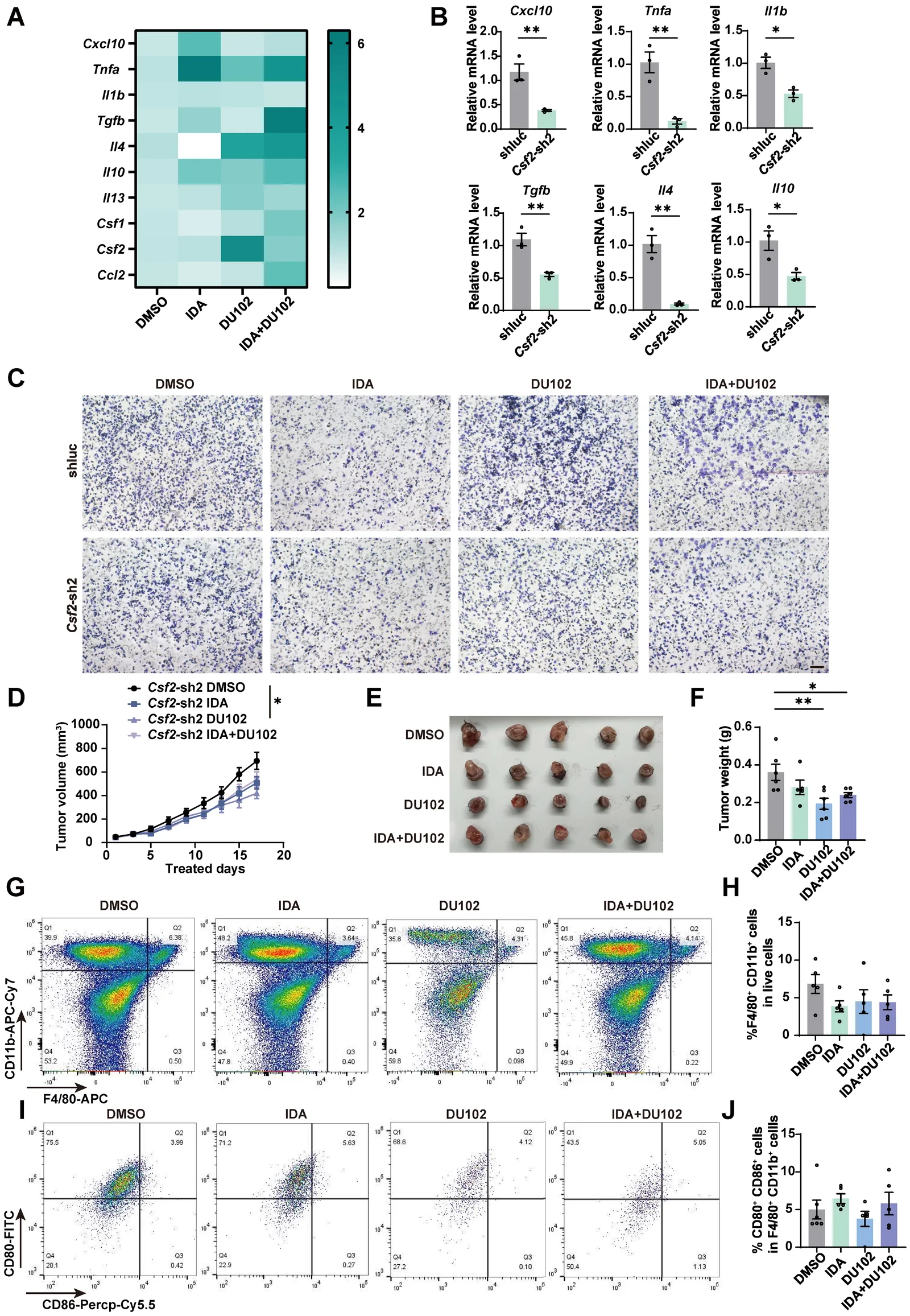
** DU102 induces CSF2 expression in tumor cells to promote macrophage expansion, which is essential for the anti-tumor effect of its combination with IDA. (A)** qPCR analysis of macrophage-related factors in Panc02 cells pretreated with DMSO, IDA (50μM), DU102 (5μM), or their combination. Data were normalized to *18S* and are presented as fold change relative to the DMSO group (n=3). **(B)** qPCR analysis of macrophage activation markers in Panc02-sh*Csf2* cells. Data are normalized to *18S* (n=3).** (C)** Transwell migration assay of RAW264.7 cells towards CM from pretreated Panc02-shluc or Panc02-sh*Csf2* cells as described in (A). Scale bar: 100 µm.** (D-F)** C57BL/6 mice bearing subcutaneous Panc02-sh*Csf2* xenografts were treated with DMSO, IDA (50mg/kg), DU102(5mg/kg), or their combination (n=5). **(D)** Tumor growth curves, **(E)** final tumor volume and** (F)** tumor weight at the study endpoint are shown.** (G-J)** Flow cytometric analysis of tumor-infiltrating macrophages cells from Panc02 allografts (from D-F) (n=5). **(G)**Representative flow cytometry plots and **(H)**quantification of F4/80⁺CD11b⁺ macrophages in live cells. **(I)**Representative flow cytometry plots and **(J)**quantification of CD80⁺CD86⁺ cells within the F4/80⁺CD11b⁺ macrophage population. Data are presented as mean ± SEM. Statistical significance was determined by two-way ANOVA with Tukey's multiple comparisons test (D), one-way ANOVA with Tukey's multiple comparisons test (F, H, J), or unpaired two-tailed t-test (B). Only significant comparisons are indicated by asterisks (*p < 0.05; **p < 0.01; ***p < 0.001; ****p < 0.0001); non-significant comparisons are not indicated.

**Figure 6:**
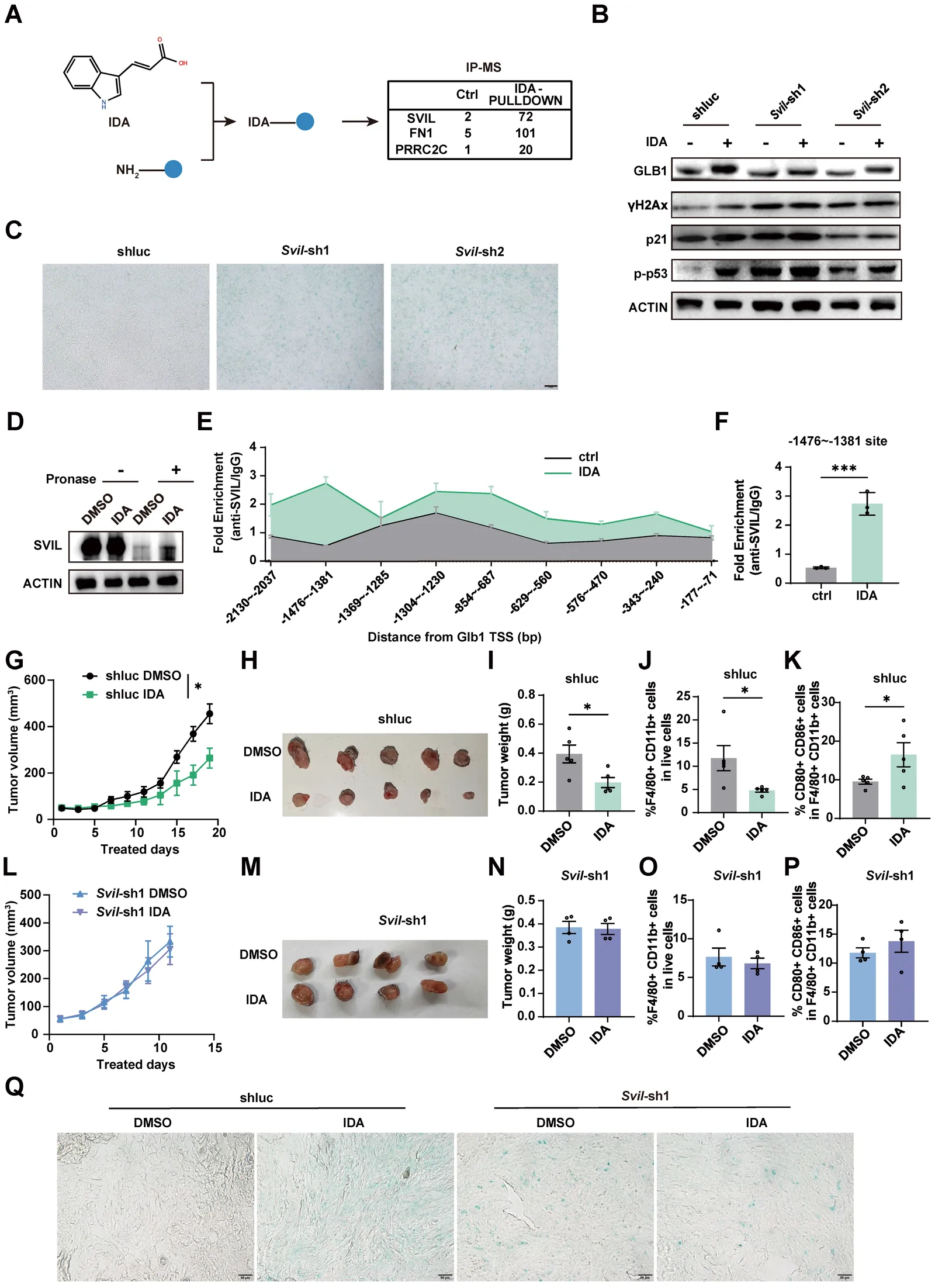
** IDA induces senescence by targeting SVIL. (A)** Schematic of the immunoprecipitation-mass spectrometry (IP-MS) approach to identify IDA-binding proteins.** (B)** Western blot analysis of senescence-associated markers in Panc02-shluc and Panc02-sh*Svil* cells. **(C)** SA-β-gal staining of Panc02-shluc and Panc02-sh*Svil* cells. Scale bar, 1mm. **(D)** DARTS assay for IDA-SVIL interaction. SVIL-FLAG protein was incubated with IDA (15mM) or DMSO, followed by limited proteolysis with thermolysin. Samples were analyzed by Western blot. Actin served as a loading control.** (E)** ChIP-qPCR analysis of SVIL enrichment on the *Glb1* promoter in Panc02 cells treated with or without IDA. Immunoprecipitation was performed using an anti-SVIL antibody or control IgG. Data are presented as fold enrichment over IgG (n=3).** (F)** Quantification of the ChIP-qPCR enrichment at the most responsive site from (E). **(G-K)** C57BL/6 mice bearing subcutaneous Panc02-shluc xenografts were treated with DMSO or IDA (50mg/kg) (n=5). **(G)** Tumor growth curves. **(H)** final tumor volume.** (I)** tumor weight at the study endpoint. **(J)** Quantification of tumor-infiltrating F4/80⁺CD11b⁺ macrophages as a percentage of live cells. **(K)** Quantification of CD80⁺CD86⁺ cells within the F4/80⁺CD11b⁺ macrophage population. **(L-P)** C57BL/6 mice bearing subcutaneous Panc02-sh*Svil* xenografts were treated with DMSO or IDA(n=4). **(L)** Tumor growth curves. **(M)** final tumor volume.** (N)** tumor weight at the study endpoint. **(O)** Quantification of tumor-infiltrating F4/80⁺CD11b⁺ macrophages as a percentage of live cells. **(P)** Quantification of CD80⁺CD86⁺ cells within the F4/80⁺CD11b⁺ macrophage population.** (Q)** SA-β-gal staining of tumor sections from (G-P). Scale bar, 50 μm. Data are presented as mean ± SEM. Statistical significance was determined by unpaired two-tailed t-test (F-G, I-L, N-P). Only significant comparisons are indicated by asterisks (*p < 0.05; **p < 0.01; ***p < 0.001; ****p < 0.0001); non-significant comparisons are not indicated.

**Figure 7:**
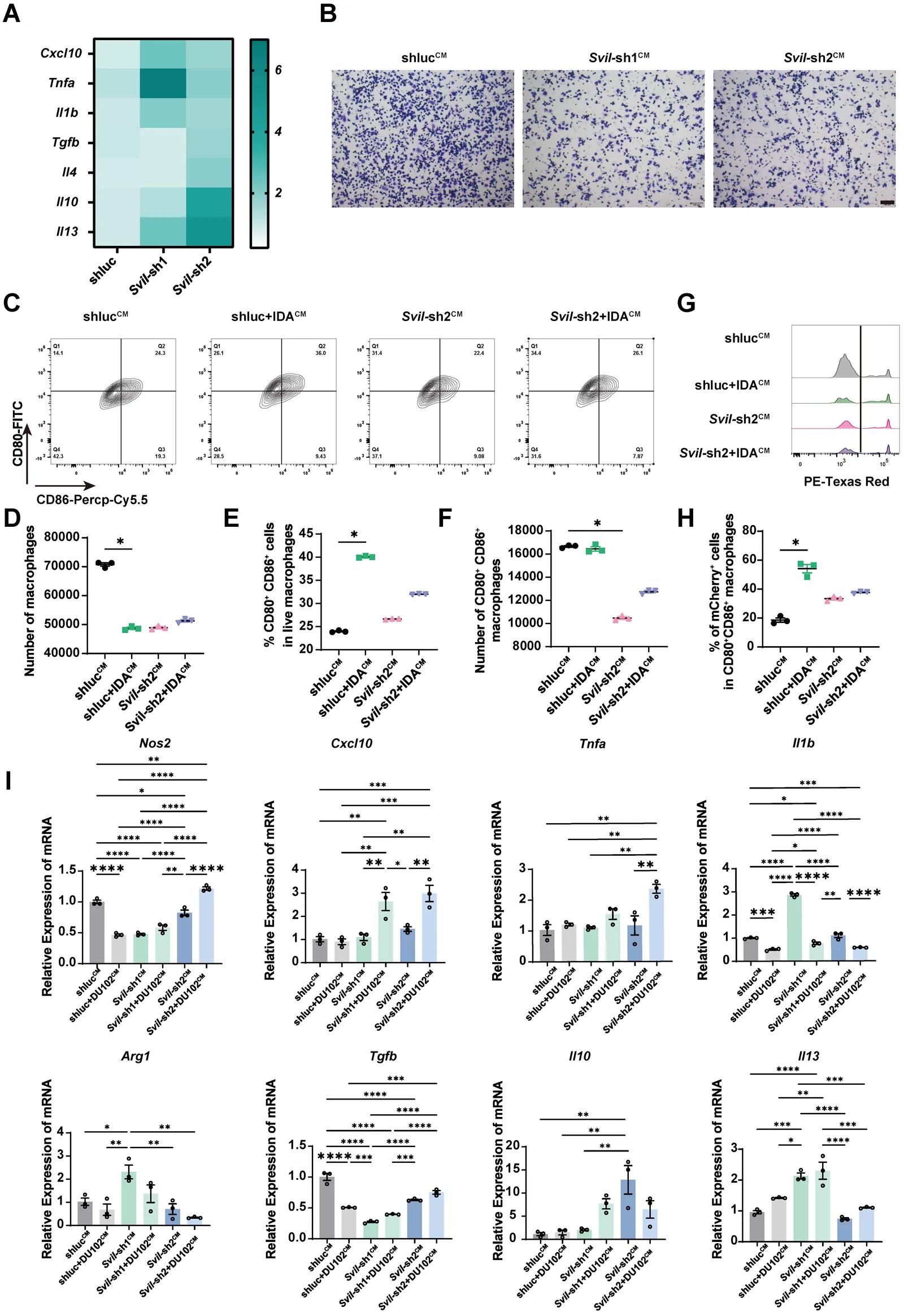
** IDA targets SVIL to induce macrophage polarization. (A)** qPCR analysis of macrophage activation-related cytokines in Panc02-shluc (control) and Panc02-sh*Svil* cells. Data were normalized to *18S* and are presented as fold change relative to the Panc02-shluc controls (n=3). **(B)** Transwell migration assay of RAW264.7 cells towards pretreated Panc02-shluc or Panc02-shSVIL cells seeded in the lower chamber. Scale bar: 100 µm.** (C-F)** Flow cytometric analysis of RAW264.7 cells for 72 hours with CM from Panc02-shluc or Panc02-sh*Svil* cells pretreated with DMSO or IDA (50μM) (n=3). **(C)** Representative flow cytometric plots of CD80⁺CD86⁺ macrophages. **(D)** Total cell counts of macrophages.** (E)** Proportion and **(F)** absolute number of CD80⁺CD86⁺ cells within F4/80⁺CD11b⁺ macrophages.** (G-H)** Phagocytosis assay. RAW264.7 macrophages were pretreated with the indicated CM for 72 h, then co-cultured with mCherry-expressing CT26 tumor cells for 3 h. **(G)** Representative flow cytometry histogram of mCherry fluorescence within the CD11b⁺F4/80⁺CD80⁺CD86⁺ macrophage gate. **(H)** Quantification of the percentage of mCherry⁺ cells within the CD11b⁺F4/80⁺CD80⁺CD86⁺ macrophage population (n=3).** (I)** qPCR analysis of macrophage activation markers in RAW264.7 cells treated with CM from Panc02-shluc or Panc02-sh*Svil* cells pre-treated with DMSO or DU102 (5 μM). Data are normalized to *Gapdh* and presented as fold change relative to the DMSO-CM group (n=3). Data are presented as mean ± SEM. Statistical significance was determined by one-way ANOVA with Kruskal-Wallis and Dunn's multiple comparisons test (D-F, H) or one-way ANOVA with Tukey's multiple comparisons test (I). Only significant comparisons are indicated by asterisks (*p < 0.05; **p < 0.01; ***p < 0.001; ****p < 0.0001); non-significant comparisons are not indicated.

**Table 1:** Key resources table

REAGENTS or RESOURCE	SOURCE	IDENTIFIER
Antibodies		
APC anti-mouse F4/80 Antibody	Biolegend	Cat# 123116; RRID: AB_893481;
APC/Cyanine7 anti-mouse/human CD11b Antibody	Biolegend	Cat# 101225; RRID: AB_830641;
FITC anti-mouse CD80 Antibody	Biolegend	Cat# 104706; RRID: AB_313127;
PerCP/Cyanine5.5 anti-mouse CD86 Antibody	Biolegend	Cat# 105028; RRID: AB_2074994;
Brilliant Violet 421™ anti-mouse/human CD11b Antibody	Biolegend	Cat# 101235; RRID: AB_10897942;
APC/Cyanine7 anti-mouse CD163 Antibody	Biolegend	Cat# 155323; RRID: AB_2936556
APC anti-mouse CD206 (MMR) Antibody	Biolegend	Cat# 141707; RRID: AB_10896057
Rabbit IgG control Polyclonal antibody	Proteintech	Cat# 30000-0-AP; RRID: AB_2819035;
anti-p-γH2Ax antibody	Proteintech	Cat# 83307-2-RR; RRID: AB_3670974;
anti-Arginase-1 antibody	Proteintech	Cat# 16001-1-AP; RRID: AB_2289842;
anti-β-actin antibody	Proteintech	Cat# 66009-1-Ig; RRID: AB_2687938;
anti-SVIL antibody	Proteintech	Cat# 27524-1-AP; RRID: AB_3669607;
Anti-p21 antibody	Proteintech	Cat# 85435-2-RR; RRID: AB_3744214;
anti-Caspase1 antibody	Abclonal	Cat# A16792; RRID: AB_2768717;
anti-Vinculin antibody	Abclonal	Cat# A2752; RRID: AB_2863020;
anti-GLB1 antibody	Abclonal	Cat#A13668; RRID: AB_2760529;
anti-iNOS antibody	Abclonal	Cat# A3774; RRID: AB_3094627;
anti-p-P53 antibody	Abclonal	Cat# AP1504; RRID: NA;
anti-CD68 antibody	Cell Signaling Technology	Cat# 97778; RRID: AB_2928056;
anti-p-TBK1 antibody	Cell Signaling Technology	Cat# 5483; RRID: AB_10693472;
anti-IL1 beta antibody	GeneTex	Cat# GTX74034; RRID: AB_378141;
Bacterial and virus strains		
Trans5α	Transgen	Cat# CD201-01
Ad-Cre	Signagen	Cat# SL100707
Primers		
Primers	Table S1	N/A
Chemicals, peptides, and recombinant proteins		
DAPI	Sigma-Aldrich	Cat# D9542
LPS	Sigma-Aldrich	Cat# L2280
Diphtheria Toxin	Sigma-Aldrich	Cat# D0564
Citrate buffer	Sigma-Aldrich	Cat# C9999
Metronidazole	MCE	Cat# HY-B0318
Neomycin sulfate	MCE	Cat# HY-B0470
Trans-3-Indoleacrylic acid	MCE	Cat# HY-W015273A
Etoposide	MCE	Cat# HY-13629
Bleomycin	MCE	Cat# HY-108345
AntiFade Mounting Medium	MCE	Cat# HY-K1042
Puromycin Dihydrochloride	Beyotime	Cat# ST551
BioPremium	Beyotime	Cat# ST1177
RBC Lysis Buffer	Beyotime	Cat# C3702
RIPA Lysis Buffer	Beyotime	Cat# P0013B
Crystal Violet Saturated Methanolic Solution	Solarbio	Cat# G1071
Neutralbalsam	Solarbio	Cat# G8590
Tissue-Tek^®^ O.C.T. Compound	SAKURA	Cat# 4583
Cell Staining Buffer	4A biotech	Cat# FXP005
Critical commercial assays		
SPlink Detection Kit	ZSGB-BIO	Cat# SP-9001
DAB kit	ZSGB-BIO	Cat# ZLI-9018
Cell Counting Kit-8	Beyotime	Cat# C0038
Senescence β-Galactosidase Staining Kit	Beyotime	Cat# C0602
Hematoxylin and Eosin Staining Kit	Beyotime	Cat# C0105S
ChIP Assay Kit	Beyotime	Cat# P2078
NovoScript®Plus All-in-one 1st Strand cDNA Synthesis SuperMix (gDNA Purge)	Novoprotein	Cat# E047-01A
ChamQ SYBR qPCR Master Mix	Vazyme	Cat# Q311-02
Experimental models: Cell lines		
HEK 293T/17	ATCC	Cat# CRL-11268™
Panc02 LLC1 Hepa 1-6	Cytion ATCC ATCC	Cat# 300501 Cat# CRL-1642™ Cat# CRL-1830 ™
A549	ATCC	Cat# CCL-185™
Panc1	ATCC	Cat# CRL-1496™
Experimental models: Organisms/strains		
Mouse: C57BL/6J	GemPharmatech	Strain #: N00013
Mouse: NOD/ShiLtJGpt-Prkdc^em26Cd52^Il2rg^em26Cd22^Hr^em1Cin8936^/Gpt mice (NCG)	GemPharmatech	Strain #: T003257
Mouse: B6.129P2-*Lyz2^tm1(cre)Ifo^*/J	The Jackson Laboratory	Strain #: 004781
Mouse: C57BL/6-*Gt(ROSA)26Sor^tm1(HBEGF)Awai^*/J	The Jackson Laboratory	Strain #: 007900
Mouse: B6.129S4-*Kras^tm4Tyj^*/J	The Jackson Laboratory	Strain #: 008179
Mouse: B6.129P2-*Trp53^tm1Brn^*/J	The Jackson Laboratory	Strain #: 008462
Mouse: B6;129-*Gt* (*ROSA*) *26Sor^tm1^*^(CAG-cas9*,-EGFP)*Fezh*^/J	The Jackson Laboratory	Strain #: 024857
Recombinant DNA		
Plko.1-P21-mCherry- puromycin	This paper	N/A
Plko.1-sh*Luciferase*-puromycin	This paper	N/A
Plko.1-sh*Svil*-1-puromycin	This paper	N/A
Plko.1-sh*Svil*-2-puromycin	This paper	N/A
Plko.1-sh*Csf2*-1-puromycin	This paper	N/A
Plko.1-sh*Csf2*-2-puromycin	This paper	N/A
Plko.1-sh*Tnfα*-1-puromycin	This paper	N/A
Plko.1-sh*Tnfα*-2-puromycin	This paper	N/A
Plko.1-sh*Tnfα*-3-puromycin	This paper	N/A
Plko.1-sh*Prrc2c*-1-puromycin	This paper	N/A
Plko.1- sh*Prrc2c* -2-puromycin	This paper	N/A
Plko.1- sh*Prrc2c* -3-puromycin	This paper	N/A
Plko.1-sh*Fn1*-1-puromycin	This paper	N/A
Plko.1- sh* Fn1* -2-puromycin	This paper	N/A
Software and algorithms		
FlowJo_V10	Becton Dickinson	https://www.flowjo.com/solutions/flowjo
GraphPad Prism 9.0.0	GraphPad	https://www.graphpad.com/
Biorender	Biorender	https://www.biorender.com/
GEPIA2	GEPIA2	http://gepia2.cancer-pku.cn/#index
CompuSyn software	ComboSyn Inc.	https://www.combosyn.com/
FreeChemDraw	FreeChemDraw	https://www.freechemdraw.com/
ZEN lite	ZEISS	https://www.zeiss.com

## Data Availability

The data that support the findings of this study are available from the corresponding author upon reasonable request.
