# Supplementary material for: The Microbial Metabolite IDA Sensitizes Tumor Cells to ARF1 Inhibition by Inducing SVIL-dependent Senescence
Source: Int J Biol Sci. 2026 Aug 24;22(14):7585–605. doi: 10.7150/ijbs.133716 (PMC13617651; doi:10.7150/ijbs.133716)
Supplement: Supplementary file 1 [file ijbsv22p7585s1.pdf]

Supplemental file for

The Microbial Metabolite IDA Sensitizes Tumor Cells to ARF1 Inhibition by Inducing SVIL-dependent Senescence

Menglin Huang[1, 2], Xuan Wang[1, 2], Yanfeng Jiang[1], Jincheng Li[1], Binliu Hu[1], Yuetong Wang[1, *], Steven X. Hou[1, *]

[1] Department of Cell and Developmental Biology at School of Life Sciences, State Key Laboratory of Genetics and Development of Complex Phenotypes, Institute of Metabolism and Integrative Biology, Zhongshan Hospital, Fudan University, Shanghai 200438, China

[2]These authors made equal contributions

*Lead contact

Correspondence: stevenhou@fudan.edu.cn (S.X.H.)

wangyt@fudan.edu.cn（Y.T.W.）

Key words

Microbial metabolite, Cellular senescence, Trans-3-Indoleacrylic acid, ARF1 inhibitor, M1-like macrophage

Figure S1. Senescence elevates cell sensitivity to DU102.

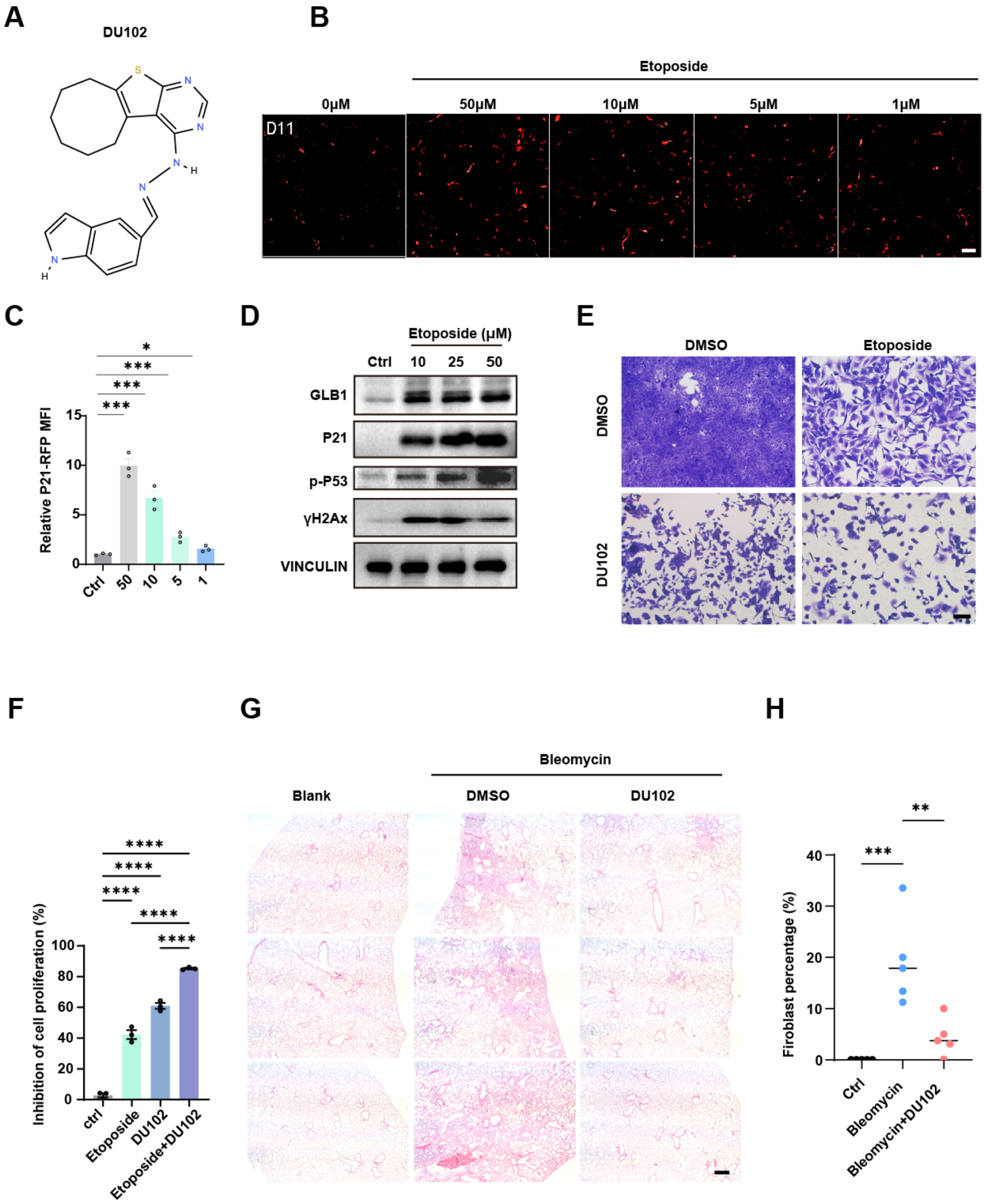

Figure S1. Senescence elevates cell sensitivity to DU102.

(A) The chemical structure of DU102. **(B)** Representative fluorescence microscopy images of Panc02 p21-RFP reporter cells treated with the indicated concentrations of etoposide for 24 h. Scale bar, 200μm. **(C)** Quantitative of mean fluorescence intensity (MFI) from (A) as a function of etoposide concentration (n=3). **(D)** Western blot analysis of senescence markers in Panc02 cells treated with increasing concentrations of etoposide (10, 25, and 50 μM) for 24 h. VINCULIN served as a loading control. **(E)** Crystal Violet staining of

Panc02 cells treated with etoposide alone or in combination with DU102 (5µM) for 24 h. **(F)** Quantification of cell viability (n=3). Scale bar, 100µm. **(G-H)** Analysis of fibrosis in KP orthotopic lung tumor model. **(G)** Representative Masson's Trichrome-stained lung sections. Blue staining indicates collagen deposition (fibrosis). **(H)** Quantification of the fibroblast area percentage (n=5). Scale bar, 50µm.

Data in all panels are presented as mean ±SEM. Statistical significance was determined by one-way ANOVA with Tukey's multiple comparisons test (C, F, G). Only significant comparisons are indicated by asterisks (*$p < 0.05$; **$p < 0.01$; ***$p < 0.001$; ****$p < 0.0001$); non-significant comparisons are not indicated.

Figure S2. Trans-3-Indoleacrylic acid is a potential senescence inducer and enhances DU102 efficacy.

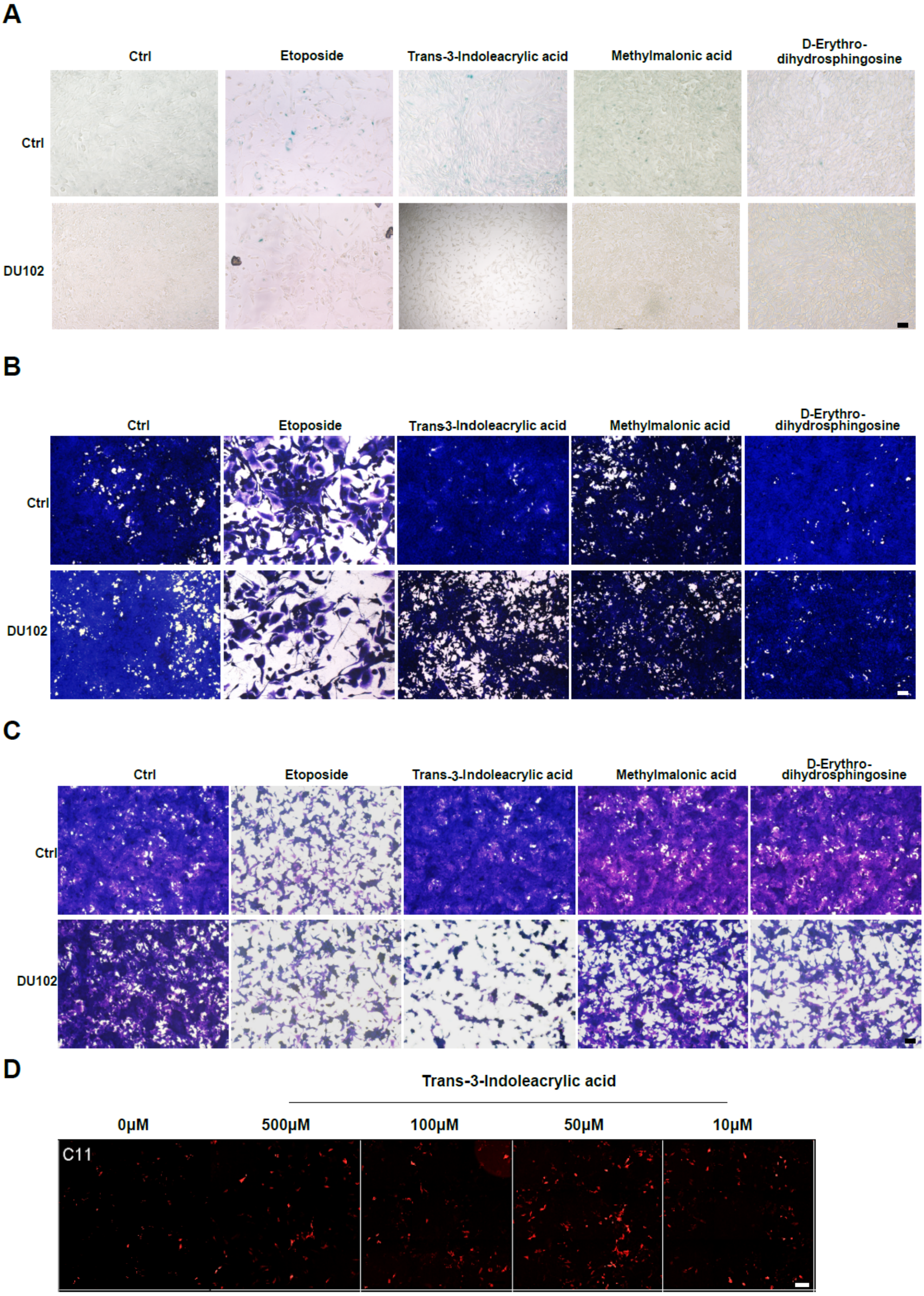

Figure S2. Trans-3-Indoleacrylic acid is a potential senescence inducer and enhances DU102 efficacy.

(A) SA-β-gal staining in panc02 cells treated with the candidate hits identified from the primary screen in Fig. 1B. Scale bar, 100μm. **(B)** Panc02 and **(C)** Hepa1-6 cells were treated with the candidates alone or in combination with DU102 (5μM) for 24 hours, followed by Crystal Violet staining to visualize viable cells. Scale bar, 100μm. **(D)**Representative fluorescence microscopy images of Panc02 p21-RFP reporter cells treated with the indicated concentrations of IDA for 24 h.

Figure S3 IDA-induced senescence sensitizes tumor cells to DU102.

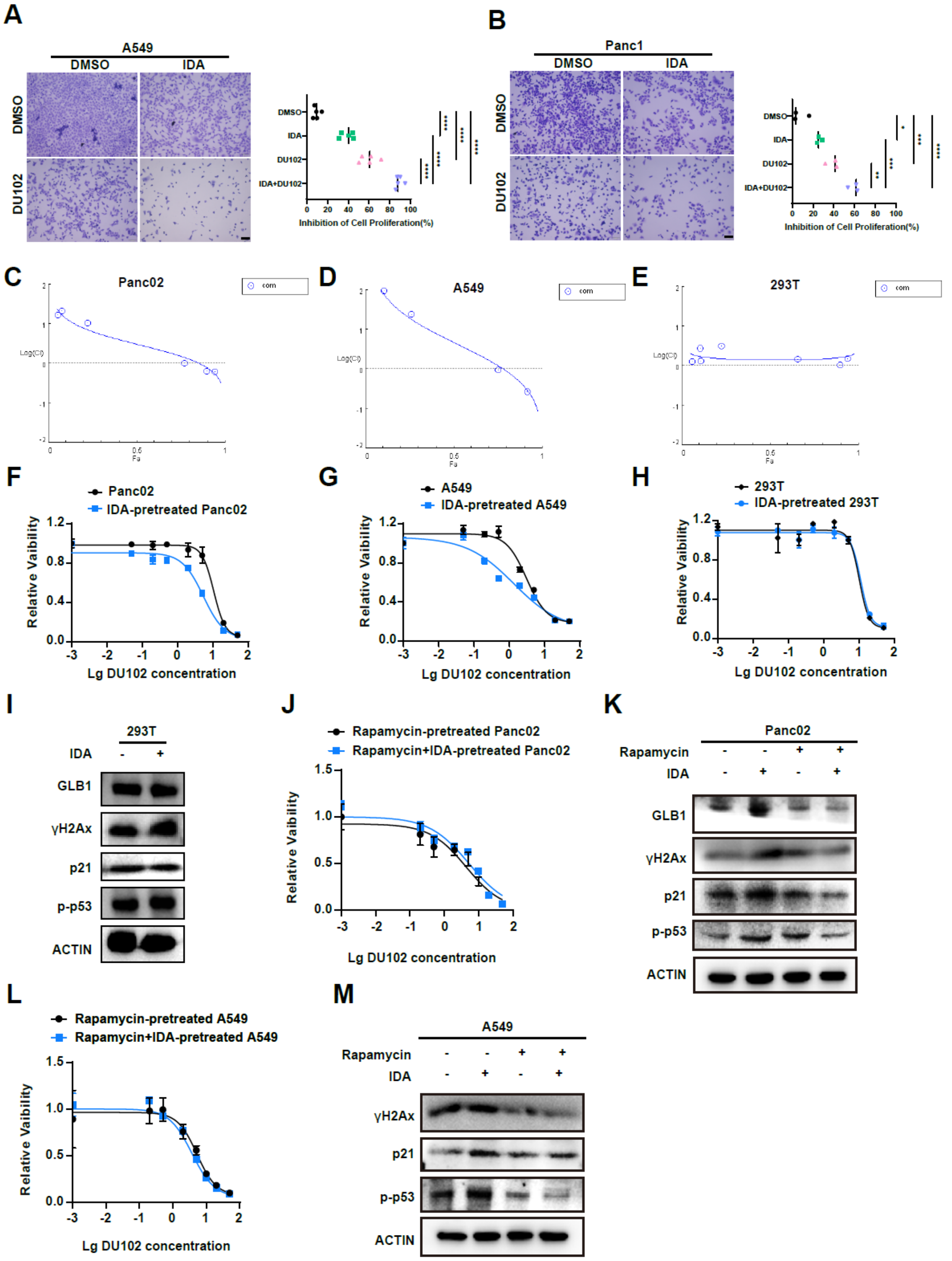

Figure S3 IDA-induced senescence sensitizes tumor cells to DU102.

(A) Crystal violet staining of A549 cells treated with DMSO, IDA (50 μM), DU102 (5 μM), or their combination (n=5). **(B)** Crystal violet staining of Panc1 cells treated as in (A) (n=3). **(C–E)** Chou-Talalay combination index (CI) analysis of IDA and DU102. Cells were treated with IDA and DU102 at a fixed ratio across a range of concentrations, and CI

values were calculated based on the fraction affected (Fa). (C) Panc02 cells. (D) A549 cells. (E) 293T cells. At senescence-inducing concentrations of IDA (0.05–0.1 mM, corresponding to Fa ≈ 0.15–0.2), the CI values were close to 1; synergy (CI < 1) was only observed at much higher IDA concentrations (≥ 26 mM, Fa ≥ 0.9). Log (CI) < 0 indicates synergy; Log (CI) > 0 indicates antagonism. **(F–H)** IDA pretreatment followed by DU102 IC50 determination. Cells were treated with IDA (50 μM) or DMSO for 5 days, after which the drug was removed, and cells were incubated with increasing concentrations of DU102 for 48–72 h. Cell viability was assessed by CCK-8 assay. **(F)** Panc02 cells. **(G)** A549 cells. **(H)** 293T cells. **(I)** Western blot analysis of senescence markers in 293T cells treated with IDA (50 μM) or DMSO for 5 days. β-ACTIN served as a loading control. **(J–M)** Rapamycin rescue experiments. Cells were treated with IDA (50 μM) and/or rapamycin (20 μM) for 5 days, followed by DU102 dose-response analysis. **(J)** DU102 dose-response curves after indicated pretreatment in Panc02 cells. **(K)** Western blot analysis of senescence markers after indicated pretreatment in Panc02 cells. **(L)** DU102 dose-response curves after indicated pretreatment in A549 cells. **(M)** Western blot analysis of senescence markers after indicated pretreatment in A549 cells. Data in all panels are presented as mean ±SEM. Statistical significance was determined by one-way ANOVA with Tukey's multiple comparisons test (A-B). Only significant comparisons are indicated by asterisks (*p < 0.05; **p < 0.01; ***p < 0.001; ****p < 0.0001); non-significant comparisons are not indicated.

Figure S4 Combination treatment suppresses tumor growth and induces senescence without overt toxicity.

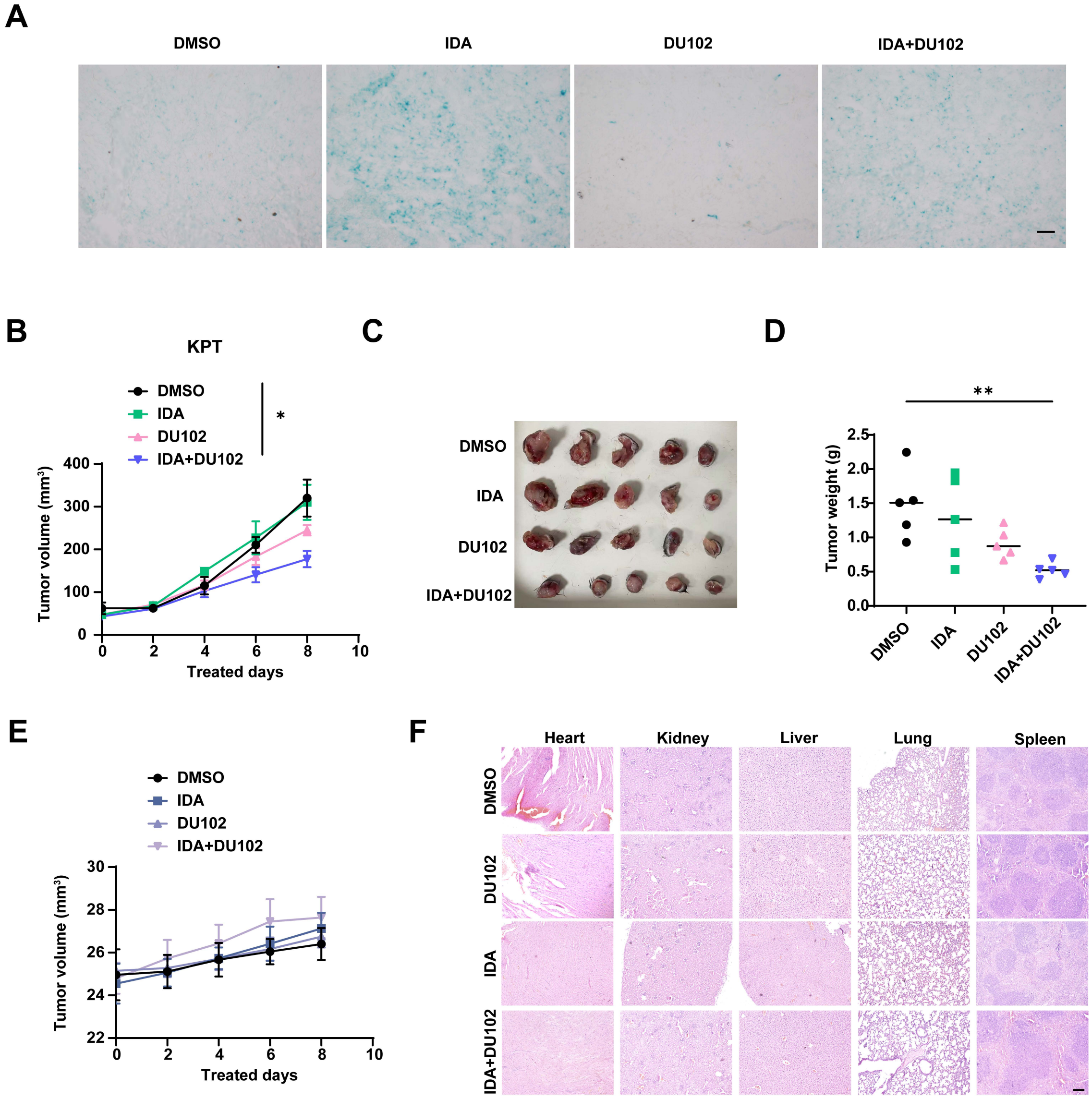

Figure S4 Combination treatment suppresses tumor growth and induces senescence without overt toxicity.

(A) SA-β-gal staining on tumor sections from Panc02 allografts in C57BL/6 mice. Scale bar, 100 μm. **(B-F)** C57BL/6 mice bearing subcutaneous KPT allografts were treated with DMSO, IDA (50 mg/kg), DU102 (5 mg/kg), or their combination (n=5). **(B)** Tumor growth curves, **(C)** final tumor volumes and **(D)** tumor weights at the study endpoint. **(E)** Body weight changes during the treatment period. **(F)** Representative H&E staining of major organs (heart, liver, spleen, lung, and kidney) from the indicated treatment groups. Scale bar, 100 μm.

Data are presented as mean ± SEM. Statistical significance was determined by Two-way ANOVA with Tukey's multiple comparisons test (B, E), or one-way ANOVA with Tukey's

multiple comparisons test (D). Only significant comparisons are indicated by asterisks (*p < 0.05; **p < 0.01; ***p < 0.001; ****p < 0.0001); non-significant comparisons are not indicated.

Figure S5 IDA modulates macrophages in the tumor microenvironment.

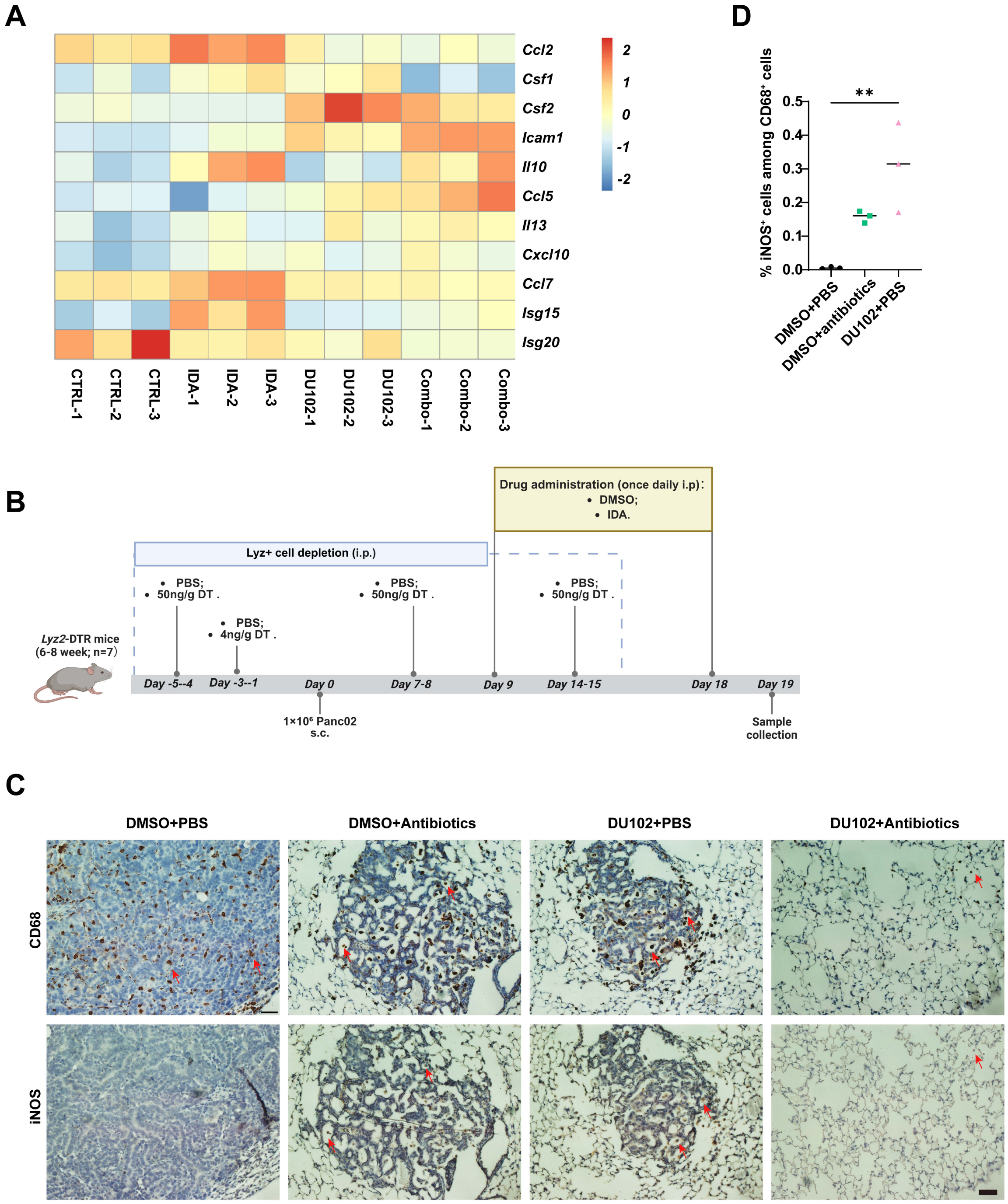

Figure S5 IDA modulates macrophages in the tumor microenvironment. Related to Figure 3.

(A) Heatmap of immune-related cytokine and chemokine mRNA expression in Panc02 cells after treatment with DMSO, IDA (50 μM), DU102 (5μM), or their combination (n=3). **(B)** Schematic of the experimental design for Lyz2+ cell depletion mediated by diphtheria toxin (DT) and subsequent drug treatment in *Lyz2*-DTR mice bearing subcutaneous Panc02 tumors. **(C)** Immunohistochemical staining for CD68 (dark brown) and iNOS (light brown) on lung sections from the orthotopic model described in

(Fig 2K), both indicated by red arrows. Scale bars, 100 μm. **(D)** Quantification of $iNOS^+$ cells among $CD68^+$ macrophages from (C) (n=3).

Data are presented as mean ±SEM. Statistical significance was determined by one-way ANOVA with Tukey's multiple comparisons test (D). Only significant comparisons are indicated by asterisks (*p < 0.05; **p < 0.01; ***p < 0.001; ****p < 0.0001); non-significant comparisons are not indicated.

Figure S6 Representative flow cytometry plots for macrophage polarization.

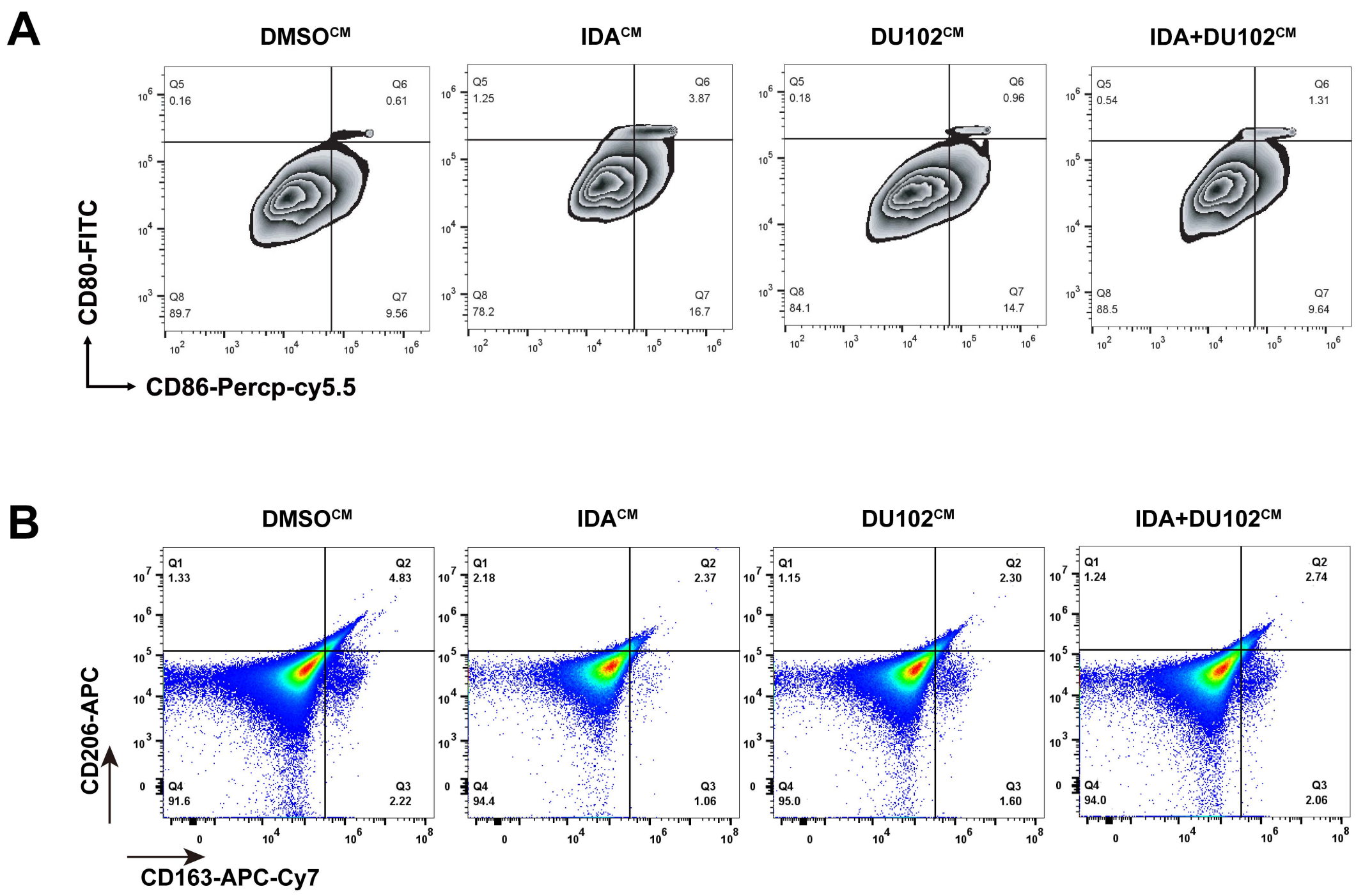

Figure S6. Representative flow cytometry plots for macrophage polarization. Related to Figure 4.

(A) Representative plots of $CD80^{+}CD86^{+}$ M1-like macrophages (corresponding to Fig. 4E). **(B)** Representative plots of $CD163^{+}CD206^{+}$ M2-like macrophages (corresponding to Fig. 4G).

Figure S7 Knockdown of *Csf2* impairs the response to IDA.

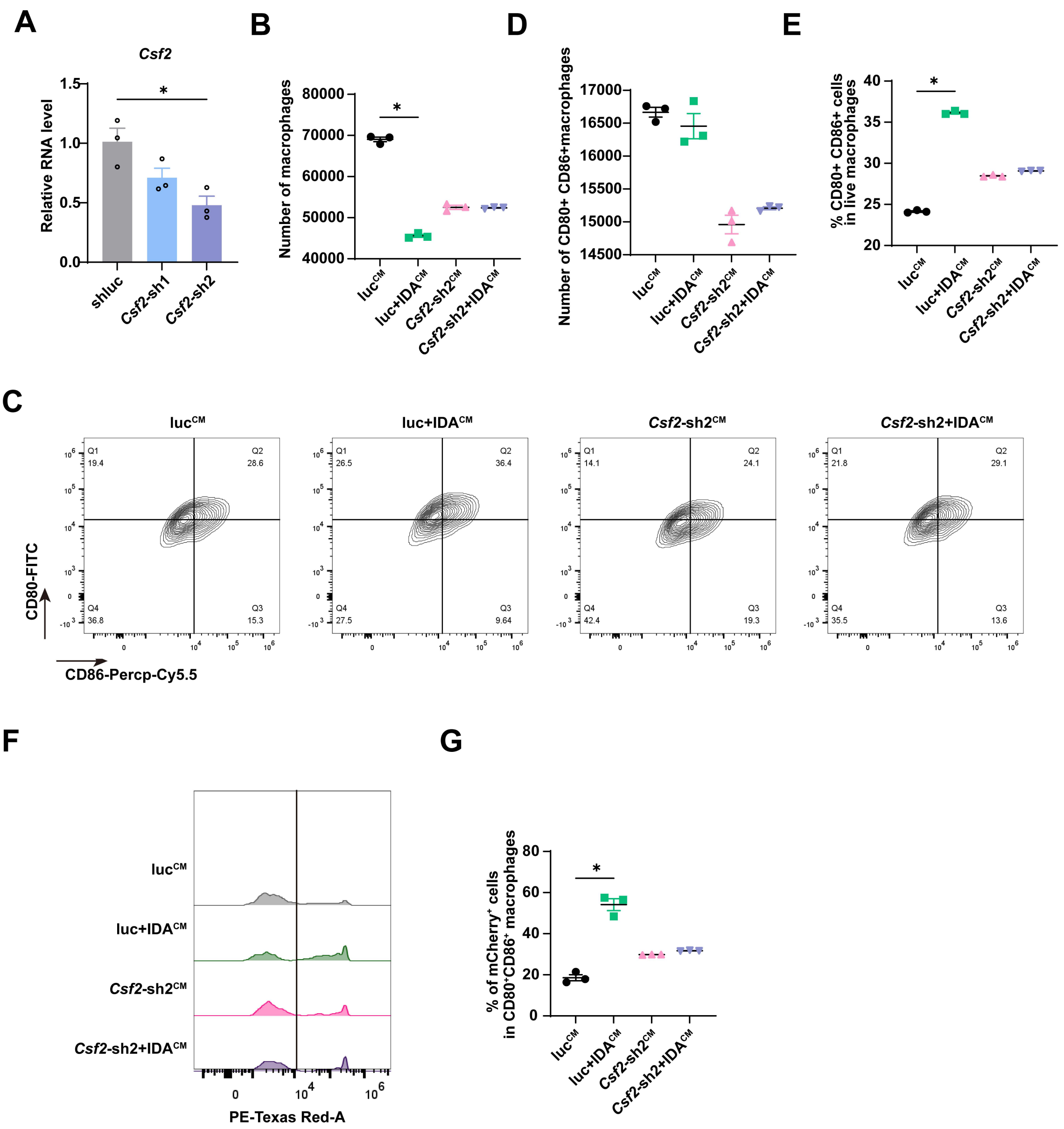

Figure S7. Knockdown of *Csf2* impairs the response to IDA. Related to Figure 5.

(A) qPCR analysis validating the knockdown efficiency of *Csf2* in Panc02 cells. Data are normalized to *18S* rRNA (n=3). **(B-E)** Flow cytometric analysis of RAW264.7 cells cultured for 72 hours with conditioned medium (CM) from Panc02-shluc or Panc02-sh*Csf2* cells, pre-treated with or without IDA (50μM). **(B)** Total live macrophage counts. **(C)** Representative flow cytometric plots of $CD80^{+}CD86^{+}$ cells. **(D)** Proportion and **(E)** absolute number of $CD80^{+}CD86^{+}$ cells within the $F4/80^{+}CD11b^{+}$ macrophage population. **(F-G)** Phagocytosis assay. RAW264.7 macrophages were pretreated with the indicated CM for 72 h, then co-cultured with mCherry-expressing CT26 tumor cells for 3 h.

(F) Representative flow cytometry histogram of mCherry fluorescence within the $CD11b^+F4/80^+CD80^+CD86^+$ macrophage gate. **(G)** Quantification of the percentage of $mCherry^+$ cells within the $CD11b^+F4/80^+CD80^+CD86^+$ macrophage population (n=3). Data are presented as mean ± SEM. Statistical significance was determined by one-way ANOVA with Kruskal-Wallis and Dunn's multiple comparisons test (B, D, E, G). Only significant comparisons are indicated by asterisks (*p < 0.05; **p < 0.01; ***p < 0.001; ****p < 0.0001); non-significant comparisons are not indicated.

Figure S8. *Tnfa* knockdown in IDA-treated tumor cells reduces macrophage polarization

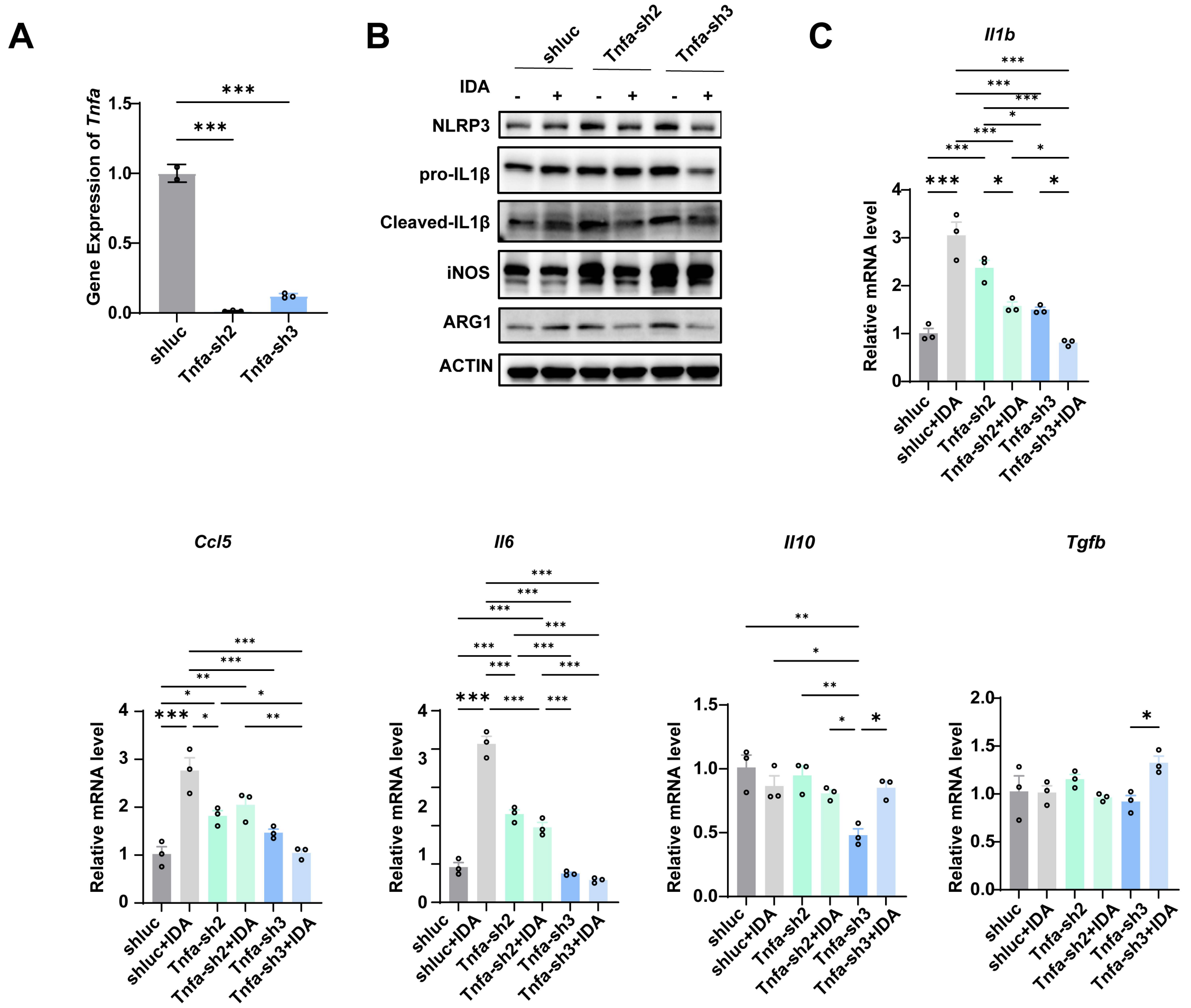

Figure S8. *Tnfa* knockdown in IDA-treated tumor cells reduces macrophage polarization. **(A)** qPCR analysis of *Tnfa* knockdown efficiency in Panc02 cells. Data are normalized to *18S*. **(B)** Western blot analysis of macrophage activation markers. β-ACTIN served as a loading control. **(C)** qPCR analysis of macrophage activation markers. Data are normalized to *18S* rRNA and presented as fold change relative to the control group (n=3). Data are presented as mean ± SEM. Data are presented as mean ± SEM. Statistical significance was determined by one-way ANOVA with Tukey's multiple comparisons test (A, C). Only significant comparisons are indicated by asterisks (*p < 0.05; **p < 0.01; ***p < 0.001; ****p < 0.0001); non-significant comparisons are not indicated.

Figure S9. SVIL is required for IDA-induced senescence.

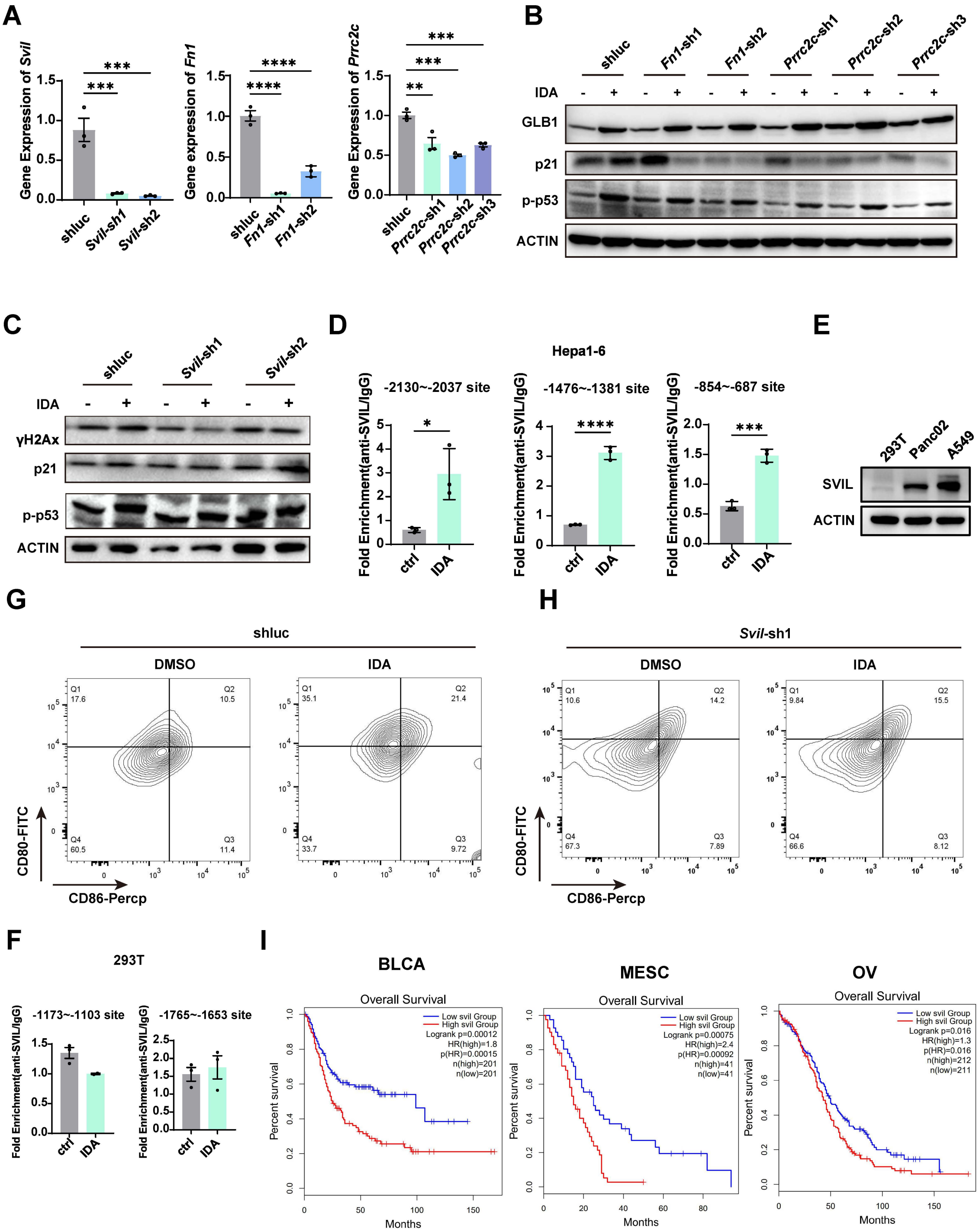

Figure S9. SVIL is required for IDA-induced senescence.

(A) qPCR analysis of *Svil*, *Fn1*, and *Prrc2c* knockdown efficiency in Panc02 cells. Data were normalized to *18S* (n=3). **(B)** Western blot analysis of senescence markers in shluc, sh*Fn1*, and sh*Prrc2c* Panc02 cells treated with IDA (50 µM) for 5 days. β-ACTIN served as a loading control. **(C)** Western blot analysis of senescence markers in A549-shluc and A549-sh*Svil* cells treated with IDA (50 µM) for 5 days. β-ACTIN served as a loading control. **(D)** ChIP-qPCR analysis of SVIL enrichment at the Glb1 promoter in

Hepa1-6 cells treated with or without IDA (50 μM) for 24 h. Immunoprecipitation was performed using an anti-SVIL antibody or control IgG. Data are presented as fold enrichment over IgG (n=3). **(E)** Western blot analysis of SVIL expression in 293T, Panc02, and A549 cells. β-ACTIN served as a loading control. **(F)** ChIP-qPCR analysis of SVIL enrichment at the *Glb1* promoter in 293T treated with or without IDA (50 μM) for 24 h. Immunoprecipitation was performed using an anti-SVIL antibody or control IgG. Data are presented as fold enrichment over IgG (n=3). **(G–H)** Representative flow cytometric plots of tumor-infiltrating macrophages in subcutaneous Panc02 xenografts. **(G)** shluc tumors treated with DMSO or IDA (corresponding to statistical quantification in Fig.6K). **(H)** sh*Svil* tumors treated with DMSO or IDA (corresponding to statistical quantification in Fig.6P). **(I)** Kaplan-Meier survival analysis of SVIL expression in cancer cohorts. High and low expression groups were stratified based on median SVIL expression. Data were analyzed using TCGA datasets. Statistical significance was determined by the log-rank test.

Data are presented as mean ± SEM. Statistical significance was determined by one-way ANOVA with Tukey's multiple comparisons test (A) or unpaired two-tailed t-test (D, F). Only significant comparisons are indicated by asterisks (*$p < 0.05$; **$p < 0.01$; ***$p < 0.001$; ****$p < 0.0001$); non-significant comparisons are not indicated.

Figure S10. Flow cytometry gating strategies.

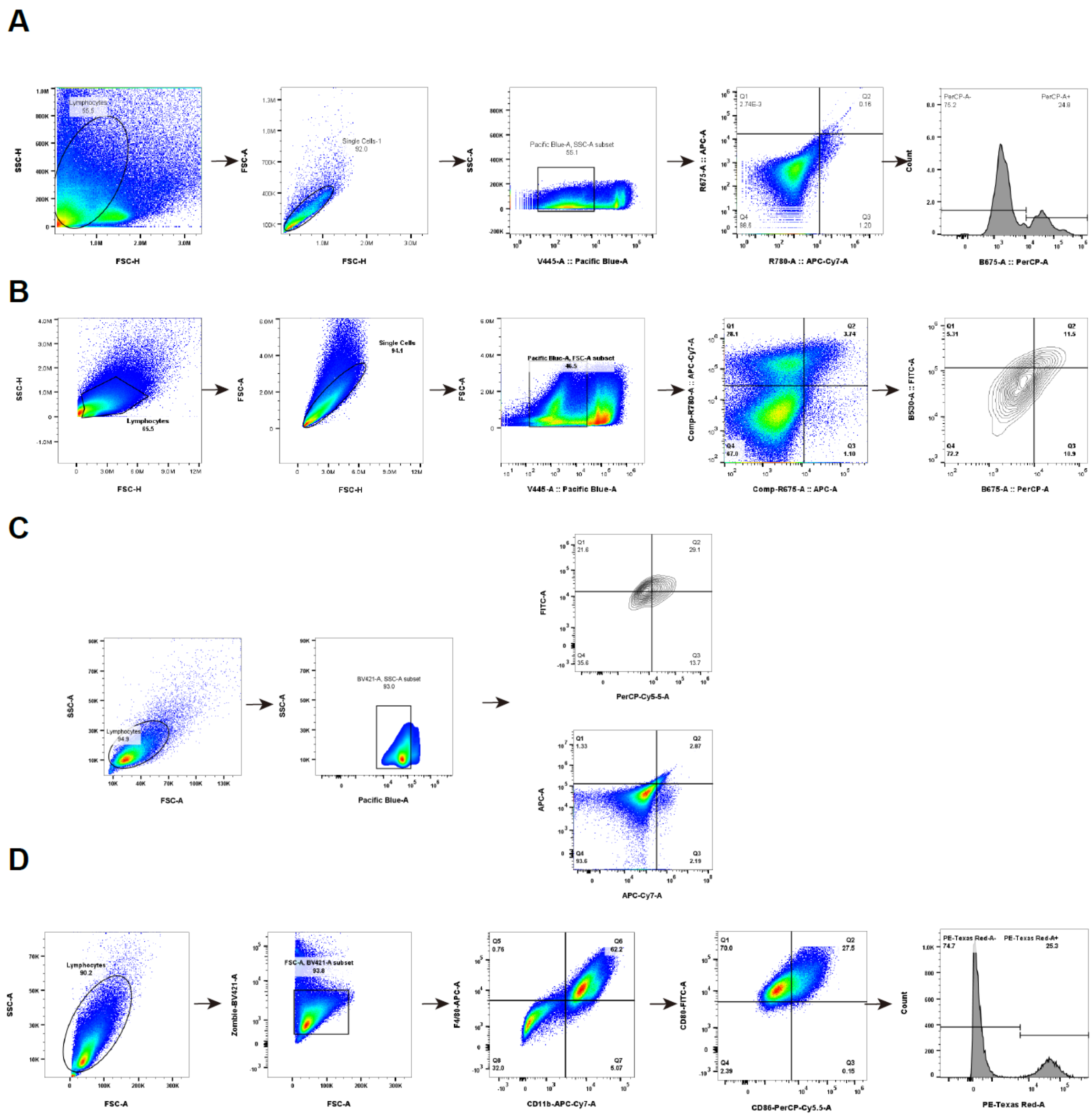

Figure S10. Flow cytometry gating strategies.
(A) Gating strategy for Lyz2-DTR mice: FSC/SSC → single cells (FSC-A/FSC-H) → live cells → CD11b^{+}F4/80^{+} macrophages → CD86 histogram. **(B)** Gating strategy for tumor-infiltrating immune cells in subcutaneous allografts: FSC/SSC → single cells (FSC-A/FSC-H) → live cells → CD11b^{+}F4/80^{+} macrophages → CD80^{+}CD86^{+} M1-like macrophages. **(C)** Gating strategy for M1/M2 polarization of RAW264.7 macrophages treated with conditioned medium (CM): FSC/SSC → live cells → CD80^{+}CD86^{+} M1-like macrophages or CD163^{+}CD206^{+} M2-like macrophages. **(D)** Gating strategy for phagocytosis assay: FSC/SSC → live macrophages → CD11b^{+}F4/80^{+} gate → CD80^{+}CD86^{+} gate → mCherry^{+} cells within the CD80^{+}CD86^{+} population.

Table S1. Primers

qPCR primer	
Name	Sequence
qp-Arg1-1F	CTCCAAGCCAAAGTCCTTAGAG
qp-Arg1-1R	AGGAGCTGTCATTAGGGACATC
qp-IL4-F	GGTCTCAACCCCCAGCTAGT
qp-IL4-R	GCCGATGATCTCTCTCAAGTGAT
qp-IL-10-2F	AGCCTTATCGGAAATGATCCAGT
qp-IL-10-2R	GGCCTTGTAGACACCTTGGT
qp-IL-6-1F	CTCCCAACAGACCTGTCTATAC
qp-IL-6-1R	CCATTGCACAACTCTTTTCTCA
M-IFNb1-q5	CCAGCTCCAAGAAAGGACGA
M-IFNb1-q3	TGGATGGCAAAGGCAGTGTA
TNFa-F-3	GTGACAAGCCTGTAGCCCAC
TNFa-R-3	TAGCAAATCGGCTGACGGTG
qp-Nos2-1F	TCCTACACCACACCAAAC
qp-Nos2-1R	CTCCAATCTCTGCCTATCC
qp-SVIL-F-1	GACACACCCAGATACATGCGT
qp-SVIL-R-1	CCCGAACTATGGATGCCTGA
qp-tgfβ1-F	CTCCCGTGGCTTCTAGTGC
qp-tgfβ1-R	GCCTTAGTTTGGACAGGATCTG
CXCL10-qF	ATGACGGGCCAGTGAGAATG
CXCL10-qR	TCAACACGTGGGCAGGATAG
Gapdh-qF	CATGGCCTTCCGTGTTCCTA
Gapdh-qR	CCTGCTTCACCACCTTCTTGAT
IL-1b-qP-F	GCTGCTTCCAAACCTTTGACC
IL-1b-qP-R	GGTGCTCATGTCCTCATCCTGG
CCL5-qF	GCTGCTTTGCCTACCTCTCC
CCL5-qR	TCGAGTGACAAACACGACTGC
ccl2-qF	CACTCACCTGCTGCTACTCA
ccl2-qR	GCTTGGTGACAAAAACTACAGC
qp-Glb1-promoter-F-1	GTGAAAGCCACCACACCACA
qp-Glb1-promoter-R-1	CTCCTGTCATCTTCTCACCTGG
qp-Glb1-promoter-F-3	GAGTCACGTGGCCTACAGAG
qp-Glb1-promoter-R-3	GTTGAGGACCCTATGCCAGG

qp-Glb1-promoter-F-4	GTGTGTGTCCTGCTCTGAGG
qp-Glb1-promoter-R-4	ACAGTGAGATGGGCGAATCC
qp-Glb1-promoter-F-5	CACTGAGGACTGACTGGCTG
qp-Glb1-promoter-R-5	TGATGAAGTTCAGGGGCTGG
qp-Glb1-promoter-F-7	GCTGTGCAGGTTCTGAGAGT
qp-Glb1-promoter-R-7	GATAGAGGGTTTGGGGTGGG
qp-Glb1-promoter-F-8	TGGCCATAGCTGAGTCACAC
qp-Glb1-promoter-R-8	CCTGAAGTCCTAGACCCGGA
qp-Glb1-promoter-F-9	CCCACCCCAAACCCTCTATC
qp-Glb1-promoter-R-9	CTGGCCTGGAACTCTAGAAGA
qp-Glb1-promoter-F-10	CCAGAGGGAAGGGCTACTGT
qp-Glb1-promoter-R-10	TGACTCAGCTATGGCCAAGTTAT
qp-Glb1-promoter-F-11	GAGGACCTGAGACTCACCG
qp-Glb1-promoter-R-1-human	CTCTCAATGAAATGTCCTTTCTGAT
qp-Glb1-promoter-F-1-human	CCTTTCTCCTTTCCTACCGGAC
qp-Glb1-promoter-R-2-human	CCAAGCTTCTTATCGTCATCATCTC
qp-Glb1-promoter-F-2-human	TGTTGGTAAAGCAGGGCTAATAA
Genotyping primer	
Lyz-Cre-F	CATATTGGCAGAACGAAAACGC
Lyz-Cre-R	CCTGTTTCACTATCCAGGTTACGG
DTR-gF1	CCTATGACCATACAACTATCCTGGC
DTR-gF2	CACTTGCTCTCCCAAAGTCGCTC
DTR-gR1	GGGTGAGCATGTCTTTAATCTACC
Cas9-F1-(WT)(5-3)	CAGGACAACGCCCACACA
Cas9-R1-(WT)(5-3	AAGGGAGCTGCAGTGGAGTA
Cas9-F2(5-3)STOPcassette	TCCCCATCAAGCTGATCC
Cas9-R2(5-3)Cas9	CTTCTTCTTTGGGGCCATCT
kras-y116-GT	TCCGAATTCAGTGACTACAGATG
kras-y117-GT	CTAGCCACCATGGCTTGAGT
kras-y118-GT	ATGTCTTTCCCCAGCACAGT
p53-GT-F	CACAAAAACAGGTTAAACCCAG

p53-GT-R	AGCACATAGGAGGCAGAGAC
shRNA primer	
shSVIL-1-F	CCGGCCTCATATTGGGCGATCCAAACTCGAGTTTGGATCGCCCAATATGAGGTTTTTG
shSVIL-1-R	AATTCAAAAACCTCATATTGGGCGATCCAAACTCGAGTTTGGATCGCCCAATATGAGG
shSVIL-2-F	CCGGTGTGACTGAGAGACGAGGAAACTCGAGTTTCCTCGTCTCTCAGTCACATTTTTG
shSVIL-2-R	AATTCAAAAATGTGACTGAGAGACGAGGAAACTCGAGTTTCCTCGTCTCTCAGTCACA
shCSF2-F-1	CCGGCGGATTTCATAGACAGCCTTACTCGAGTAAGGCTGTCTATGAAATCCG TTTTTG
shCSF2-R-1	AATTCAAAAACGGATTTCATAGACAGCCTTACTCGAGTAAGGCTGTCTATGAAATCCG
shCSF2-F-2	CCGGCGTCTCTAACGAGTTCTCCTTCTCGAGAAGGAGAACTCGTTAGAGACG TTTTTG
shCSF2-R-2	AATTCAAAAACGTCTCTAACGAGTTCTCCTTCTCGAGAAGGAGAACTCGTTAGAGACG
shTNFa-F-1	CCGGGCTATCTCATACCAGGAGAAACTCGAGTTTCTCCTGGTATGAGATAGC TTTTTG
shTNFa-R-1	AATTCAAAAAGCTATCTCATACCAGGAGAAACTCGAGTTTCTCCTGGTATGAGATAGC
shTNFa-F-2	CCGGCGATGGGTTGTACCTTGTCTACTCGAGTAGACAAGGTACAACCCATCG TTTTTG
shTNFa-R-2	AATTCAAAAACGATGGGTTGTACCTTGTCTACTCGAGTAGACAAGGTACAACCCATCG
shTNFa-F-3	CCGGCCTCCCTCTCATCAGTTCTATCTCGAGATAGAACTGATGAGAGGGAGG TTTTTG

shTNFa-R-3	AATTCAAAAACCTCCCTCTCATCAGTTCTATCTCGAGA TAGAACTGATGAGAGGGAGG
shFN1-F-1	TCTCTTCTCGAGAAGAGAAAGGTATTTCTAGGCttttttgtag aattctcgacctcgagaca
shFN1-R-1	TCTCTTCTCGAGAAGAGAAAGGTATTTCTAGGCccggtgttt cgtcctttccaca
shFN1-F-2	TATTTACTCGAGTAAATAGCTGTTCGGACATCAttttttgtag aattctcgacctcgagaca
shFN1-R-2	TATTTACTCGAGTAAATAGCTGTTCGGACATCAccggtgttt cgtcctttccaca
